# Evaluation of Apparatus and Protocols to Measure Human Passive Neck Stiffness and Range of Motion

**DOI:** 10.1007/s10439-024-03517-w

**Published:** 2024-04-24

**Authors:** Mingyue Liu, Ryan D. Quarrington, Baptiste Sandoz, William S. P. Robertson, Claire F. Jones

**Affiliations:** 1 School of Electrical and Mechanical Engineering, The University of Adelaide, Adelaide, SA Australia; 2 Adelaide Spinal Research Group, Centre for Orthopaedic & Trauma Research, Faculty of Health and Medical Sciences, The University of Adelaide, Adelaide, SA Australia; 3 Arts et Métiers Institute of Technology, Université Sorbonne Paris Nord, IBHGC – Institut de Biomécanique Humaine Georges Charpak, HESAM Université, Paris, France; 4 Department of Orthopaedics & Trauma, Royal Adelaide Hospital, Adelaide, SA Australia

**Keywords:** Cervical spine, In vivo, Motion capture, Electromyography, Method, Repeatability, Passive motion, Active motion

## Abstract

Understanding of human neck stiffness and range of motion (ROM) with minimal neck muscle activation (“passive”) is important for clinical and bioengineering applications. The aim of this study was to develop, implement, and evaluate the reliability of methods for assessing passive-lying stiffness and ROM, in six head-neck rotation directions. Six participants completed two assessment sessions. To perform passive-lying tests, the participant’s head and torso were strapped to a bending (flexion, extension, lateral bending) or a rotation (axial rotation) apparatus, and clinical bed, respectively. The head and neck were manually rotated by the researcher to the participant’s maximum ROM, to assess passive-lying stiffness. Participant-initiated (“active”) head ROM was also assessed in the apparatus, and seated. Various measures of apparatus functionality were assessed. ROM was similar for all assessment configurations in each motion direction except flexion. In each direction, passive stiffness generally increased throughout neck rotation. Within-session reliability for stiffness (ICC > 0.656) and ROM (ICC > 0.872) was acceptable, but between-session reliability was low for some motion directions, probably due to intrinsic participant factors, participant-apparatus interaction, and the relatively low participant number. Moment-angle corridors from both assessment sessions were similar, suggesting that with greater sample size, these methods may be suitable for estimating population-level corridors.

**Supplementary Information:**

The online version contains supplementary material available at 10.1007/s10439-024-03517-w.

## Introduction

A detailed understanding of six degree-of-freedom human neck stiffness and range of motion (ROM), and the potential influence of posture, muscle activation, demographics, and anthropometry on these characteristics, is important for clinical and bioengineering applications. For example, it can provide baseline data to assess biomechanical changes associated with neck pain [1, 2], whiplash [3, 4] or other neck pathologies, and provide normative data with which to design and assess surrogate necks for patient simulator mannequins and human-like robots, and to construct computational models of the human neck.

Human head-neck ROM resulting from participant-initiated motions has been relatively widely reported for healthy participants. ROM measurement modalities used in clinical and research settings have included medical imaging such as ultrasound [5–7] and fluoroscopy [8], three-dimensional motion capture [9], and physical apparatus such as the Spine Motion Analyser device [10], Cervical Range of Motion (CROM) Device [11, 12], and goniometers [13]. Assessments are typically undertaken in the upright seated or standing posture, and while good to excellent between-trial reliability has been reported for various measurement techniques [10–13], similar outcomes have not been reported in the lying posture with passive neck musculature.

Several studies have reported head-neck stiffness (with ROM) derived from tests in which a researcher applies known loads to the head to effect rotation of the head and neck relative to the stationary torso [14–16]. Such stiffness measures have generally been termed “passive”, because the motion is researcher-initiated, and muscle activity is assumed to be low or minimal compared to participant-initiated motions. McGill et al. [16] measured passive neck stiffness in flexion, extension, and lateral bending motions, while Dugailly et al. [14] measured stiffness and ROM in axial rotation. Both fixed the torso to a horizontal surface, and the head to a custom moving support independent of the torso support, in the lying position. To assess motions about the three primary anatomical axes, McClure et al. [15] strapped a linkage device to the participants’ head and torso in a seated position, but only flexion, right lateral bending and total axial rotation results were reported. In each of these studies, participants were instructed to maintain relaxed neck muscles while the head was manipulated by the researcher. Neck ROM and stiffness were derived from head-to-torso motions and the applied load. It is unclear if muscle activation remained minimal throughout the movement for all trials in previous studies, due to the lack of consistent muscle activation monitoring. None of the studies assessed stiffness and ROM in all six head rotations, or reported apparatus and test protocol factors such as friction, rotational moment, and participants’ head-torso alignment.

The aims of this study were to: (1) design and evaluate apparatus and procedures with which to assess passive neck stiffness and ROM about the three anatomical axes in the lying position; (2) assess the potential effect of these apparatus and procedures on the neck ROM achieved by comparing to active-lying and active-seated ROM; and, (3) assess the reliability of these measures between trials in a single session, and between sessions performed at least one week apart.

## Materials and Methods

Participants and testing procedures are described first to provide context, followed by a detailed description of the custom apparatus design and assessment, and data processing and statistical analysis. Procedures were approved by the institutional Human Research Ethics Committee (Approval number: H-2020-181), and participants provided written consent.

### Participants and Protocol

Participants were 20–30 years old and were in general good health. Exclusion criteria were: neck pain (last 3 months), history of vertigo, diagnosed neurological or cardiovascular disease, spinal disorders or spinal injuries. Participants were asked to refrain from alcohol intake greater than one standard drink (10 g of alcohol) for 24 h, and heavy neck or shoulder training for 72 h, prior to the test days.

**Fig. 1:**
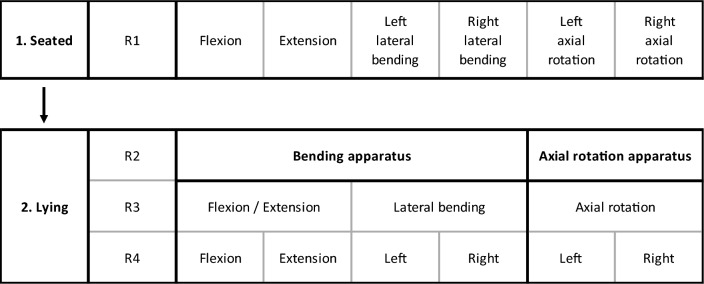
Testing protocol and motion randomisation. Seated tests were performed first; motions were assessed in random order (R1). Lying tests were performed second. The order of the apparatus (bending or axial rotation) used was randomised (R2). For the bending apparatus, the order in which flexion/extension (side-lying) or lateral bending (prone) was assessed and randomised (R3). Within each apparatus and motion configuration, the order in which direction (flexion-extension, left-right) was assessed was randomised (R4)

Each participant completed two identical testing sessions, separated by at least one week (maximum of four weeks). Six head-neck motions (flexion, extension, left/right lateral bending, left/right axial rotation) were assessed in seated and lying positions. Five trials were performed in each configuration. Seated tests to measure participant-guided (“active”) ROM, were performed first; head-neck motion order was randomised. Lying tests, to measure researcher-guided (“passive”) ROM and stiffness and participant-guided (“active”) ROM, were semi-randomised by apparatus and motion order to minimise the total duration of participant-apparatus positioning (Fig. 1). For each apparatus-motion combination, passive and active assessments were conducted chronologically.

### Marker and Electrode Placement

To measure head-neck angular displacement, reflective markers (S.1) were placed on the participant head, torso, and the apparatus (see “Bending Apparatus Design and Assessment” and “Axial Rotation Apparatus Design and Assessment” sections), and the locations of the markers were tracked with a thirteen-camera 3D motion capture system (Vantage, Vicon Motion System, UK). To define a cartesian coordinate system aligned with the Frankfort plane, markers were placed on the left and right tragion (L_T, R_T) and orbit inferior margins (L_IO, R_IO) [17] (S.2). To mitigate against marker occlusion during motion trials, the Frankfort plane marker locations were described in coordinate systems defined by a 5-marker head cluster (prone positions), or by markers placed on L_IO, L_T, and the left mastoid process (L_MP, side-lying positions). The torso coordinate system was defined by markers on C7, T8, the sternal notch (IJ), and the xiphoid process (XP) [18] (S.2). IJ, XP, and C7 movement were described in the coordinate system defined by a 4-marker cluster placed on the torso midline between the C7 and T8 markers.

Electromyography (EMG) was used to monitor neck muscle activation. Surface EMG electrodes (Trigno Mini sensors, Delsys Incorporated, USA) were placed bilaterally on the sternocleidomastoid (SCM; mid-belly, approximately one-third above the sternal attachment) [19], and splenius (SPL; 6 cm lateral from C4) [20, 21], and reference electrodes were placed inferior to the corresponding clavicle. Surface EMG electrodes (Trigno Avanti sensors, Delsys Incorporated, USA) were placed bilaterally on the trapezius (TRP, mid-point between C7 and acromion) [22] (S.1).

### Participant Assessment Procedure

Body height and mass, head girth (passing through glabella and opisthocranion) [22], and neck girth (passing through C4), were measured. Anatomical and tracking marker positions were defined in the motion capture global coordinate system in a standing neutral posture (forward gaze) with hands to the side. To familiarise them with the tasks and mobilise their neck, the participant then performed slow rotation of their head-neck, in the six assessment directions, to their maximum ROM in a standing position.

To measure participant-guided (“active”) head-neck ROM, the participant sat upright on a custom chair with their torso strapped to the chair’s back support. In each of the six directions of motion, the participant rotated their head and neck from the neutral position to their maximum range then back to the neutral position, at their preferred speed. They were directed to achieve this with minimal rotation about the other primary axes.

**Fig. 2:**
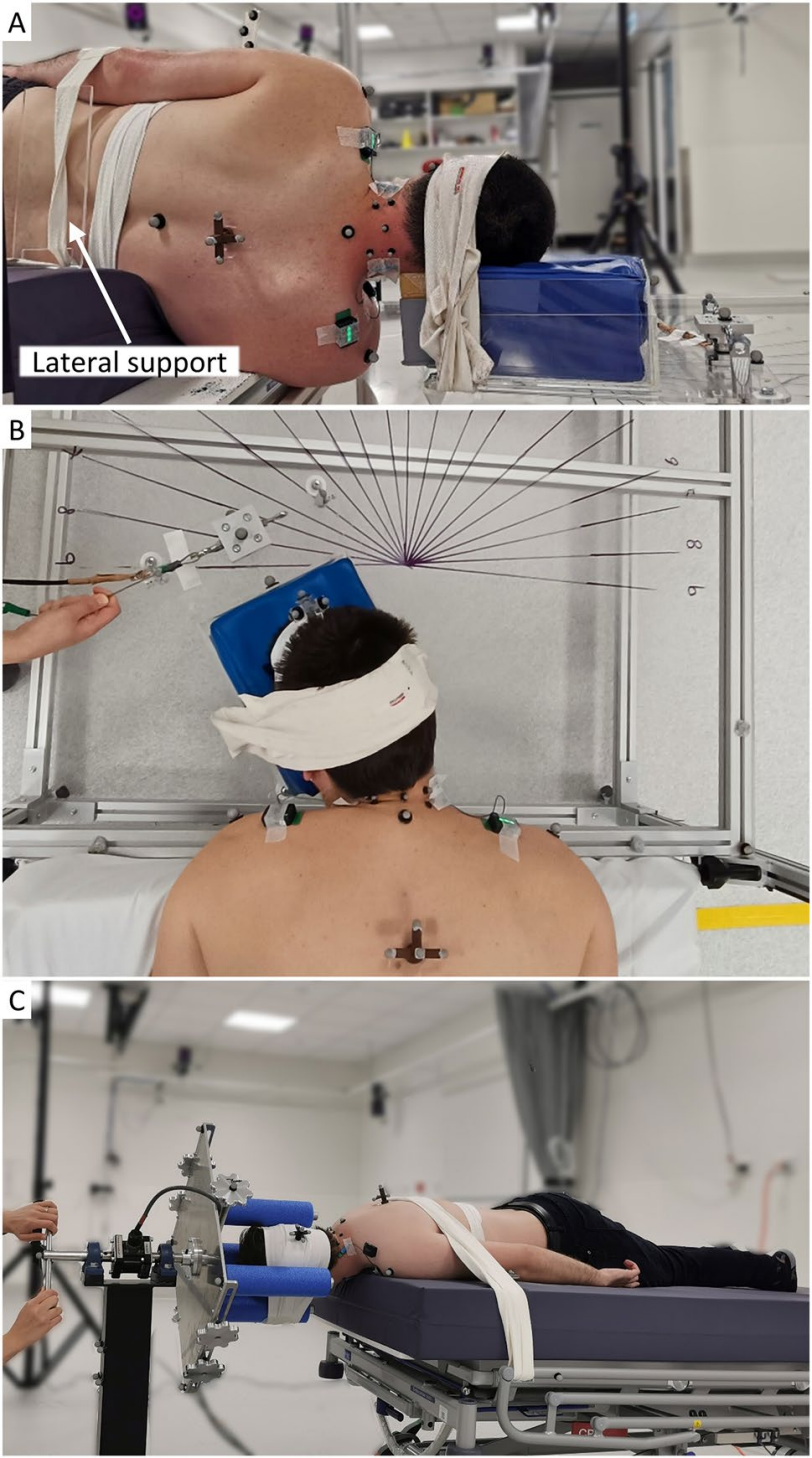
Exemplar images of the participant and apparatus. **A** Flexion/extension test with bending apparatus. Participant was strapped to the lateral support and right arm was extended. **B** Passive left lateral bending test with bending apparatus. **C** Passive right axial rotation test with rotational apparatus

For the lying tests, the participant was positioned in a neutral posture on a height- and tilt-adjustable clinical bed (Enterprise 5000X, Arjo, Australia) with their head supported by the custom apparatus (Fig. 2). The torso was strapped to the bed, and a lateral support was applied to prevent torso rotation in the side-lying position. The head was fixed to the bending or rotational apparatus with flexible straps (Fabrifoam SuperWrap, Victor, Australia). A flexible contour-tracing ruler (Art Studio Flexible Curve 60 cm, Rioti, Australia) was used to assist achieving neutral postures in the prone and side-lying positions (S.3). The ruler was conformed to the participant’s head, neck, and upper back in the neutral standing posture. In each lying position, the head and body supports were then adjusted such that the head-neck-torso posture closely matched the pre-shaped ruler. The position of all markers, in the laboratory coordinate system, was recorded in the stationary neutral posture.

Participants performed three trials of maximum voluntary contraction (MVC; 3 s contraction followed by 5 s rest) by exerting maximum effort to pull, or rotate, their head while it was constrained in the neutral position (S.4) in the respective apparatus and motion configuration. The activation signals of the agonist muscles during each MVC test (Table 1) were processed (full-wave rectified, and root-mean-square smoothed with a 200-millisecond window) immediately using a custom graphical user interface (MATLAB, R2020a, MathWorks, USA). The mean activation signal across each contraction epoch was calculated, and the highest value was defined as the MVC signal magnitude for each corresponding agonist muscle.

**Table 1:** Agonist muscles for each motion [20, 33] (*L* left, *R*: right, *SCM* sternocleidomastoid muscle, *SPL* splenius muscle)

Motions	Agonist muscles
Flexion	L_SCM and R_SCM
Extension	L_SPL and R_SPL
Left lateral bending	L_SCM and L_SPL
Right lateral bending	R_SCM and R_SPL
Left axial rotation	R_SCM and L_SPL
Right axial rotation	L_SCM and R_SPL

During the passive tests, participants were instructed to relax while their head was pulled in an arc (Fig. 2A: flexion, extension; Fig. 2B: lateral bending) or rotated (Fig. 2C: axial rotation) at approximately 10°/s, until they verbally indicated the end of their comfortable range of motion. A real-time feedback system (described further in following section “EMG Real-Time Feedback System”) was used to visually monitor EMG and audibly inform participants to relax further if the sum of agonist muscle activation exceeded 20% MVC (half of the passive threshold). While MVC value is dependent on the neck rotational angle and posture [23], defining passive threshold based on an angle-specific MVC was impractical due to the time required to measure MVC at multiple rotational angles, and the likely resultant participant fatigue. The passive threshold was defined as 40% MVC in a neutral head-neck position, because pilot testing indicated that most participants’ muscle activation remained below this value in all neck rotational directions during passive tests and remained above this level during active tests (S.5). Trials were repeated if agonist muscle activation was not consistently maintained below the threshold for the majority of the trial.

For the active-lying tests, participants were asked to rotate their head and neck at their preferred speed in each of the required directions, to their maximum ROM, then return to the initial position. To ensure static friction was overcome prior to the motion range of interest, both passive and active lying tests were commenced with the head-neck approximately 20° beyond the neutral position (i.e. in the opposite direction), which was located according to the 10° markers on the apparatus.

### Bending Apparatus Design and Assessment

**Fig. 3:**
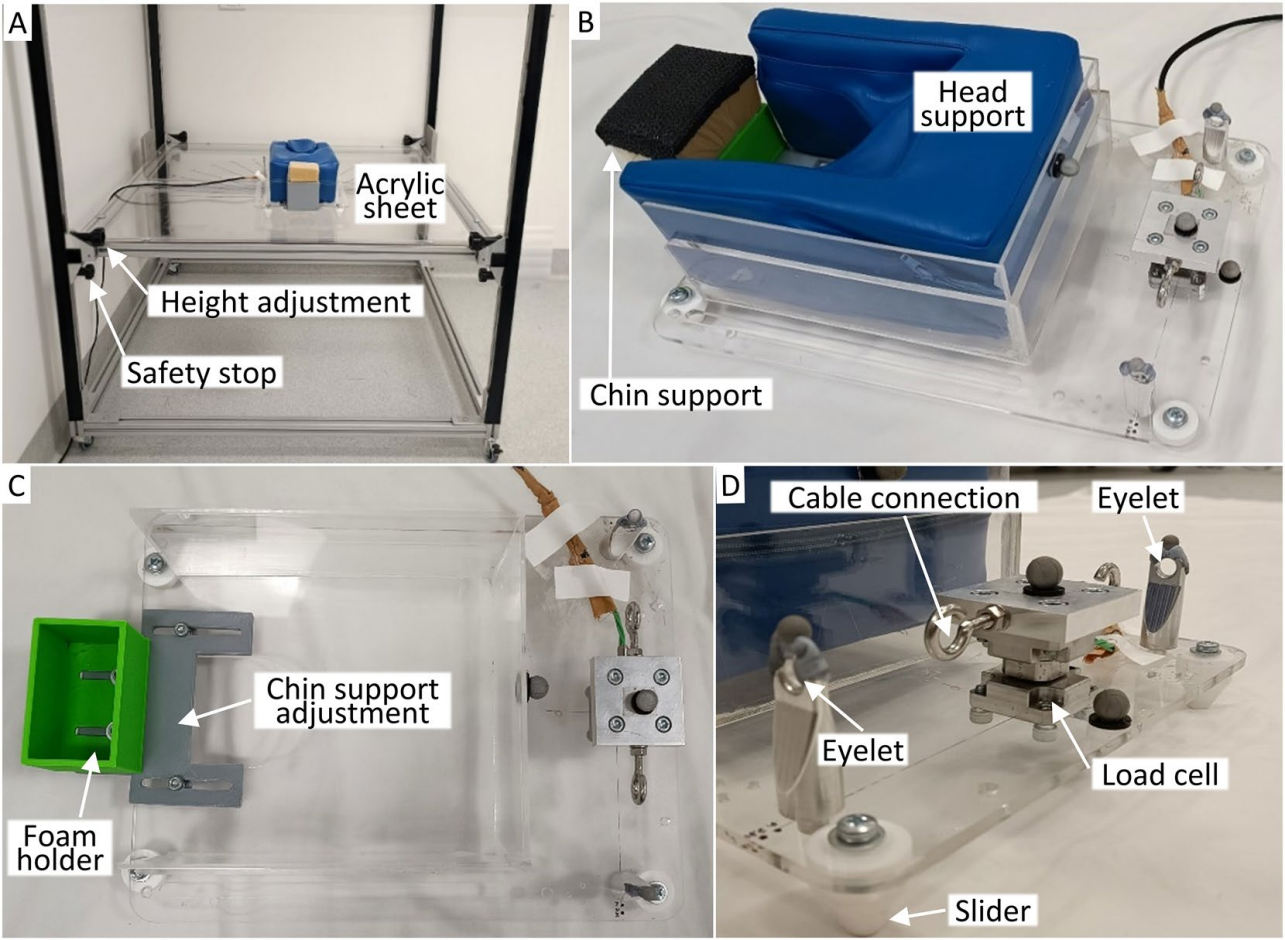
Bending apparatus. **A** Acrylic sheet mounted on height adjustable frame, with head support configured for flexion and extension tests. **B** Head support configured for lateral bending tests. A custom vinyl-covered foam cushion with rigid acrylic surrounds, supported the forehead and lateral aspect of the face without covering the mouth and nose. A separate chin support was added for the prone position. **C** Top view of head support with foam removed to show adjustable chin support and foam holder. (D) Detailed view of load cell, cable connection location, and eyelet for maintaining tangential load application (cable not shown for clarity)

The bending apparatus was used to assess flexion, extension (side-lying), and lateral bending motions (prone). The apparatus consisted of a head support which moved upon a low-friction acrylic surface mounted on a height-adjustable frame (Fig. 3A). Four polytetrafluoroethylene (PTFE) sliders mounted under the head support (Fig. 3B, C) provided low and uniform dynamic friction between the head support and the acrylic surface. Friction was further reduced by applying a plastic polish spray (Furniture and Surface Polish, Glitz, Australia) to the acrylic surface before each pair of tests (i.e. flexion and extension tests, left and right lateral bending tests). For researcher-led motions, the participant’s head-neck was pulled by the researcher via a cable fixed to a three-axis load cell (9327C, Kistler Group, Switzerland) that was mounted superior to the head on the head support (Fig. 3D). The cable passed through an eyelet, mounted on the head support laterally to the load cell; by ensuring the cable remained centrally located in the eyelet throughout motion, the applied load remained tangential to the motion path. Reflective markers were placed on top of the load cell centre and eyelets, to track cradle movement and assist stiffness calculation (details in “Data Acquisition and Post-processing”). Ten-degree markings on the acrylic surface, together with a 60 Hz metronome, were used to guide the speed with which the head-neck was pulled. Two vertical plates, with cables, fixed to the side of the frame constrained the head in a stationary position during MVC tests (S.4). The force associated with dynamic friction between the PTFE sliders on the head support (weighted with a 5 kg mass to simulate the head) and the acrylic surface, was characterised after applying the plastic polish spray, and again at the end of the participant’s assessment. Friction force was defined as the mean tangential force from three consecutive repetitions (S.6).

### Axial Rotation Apparatus Design and Assessment

**Fig. 4:**
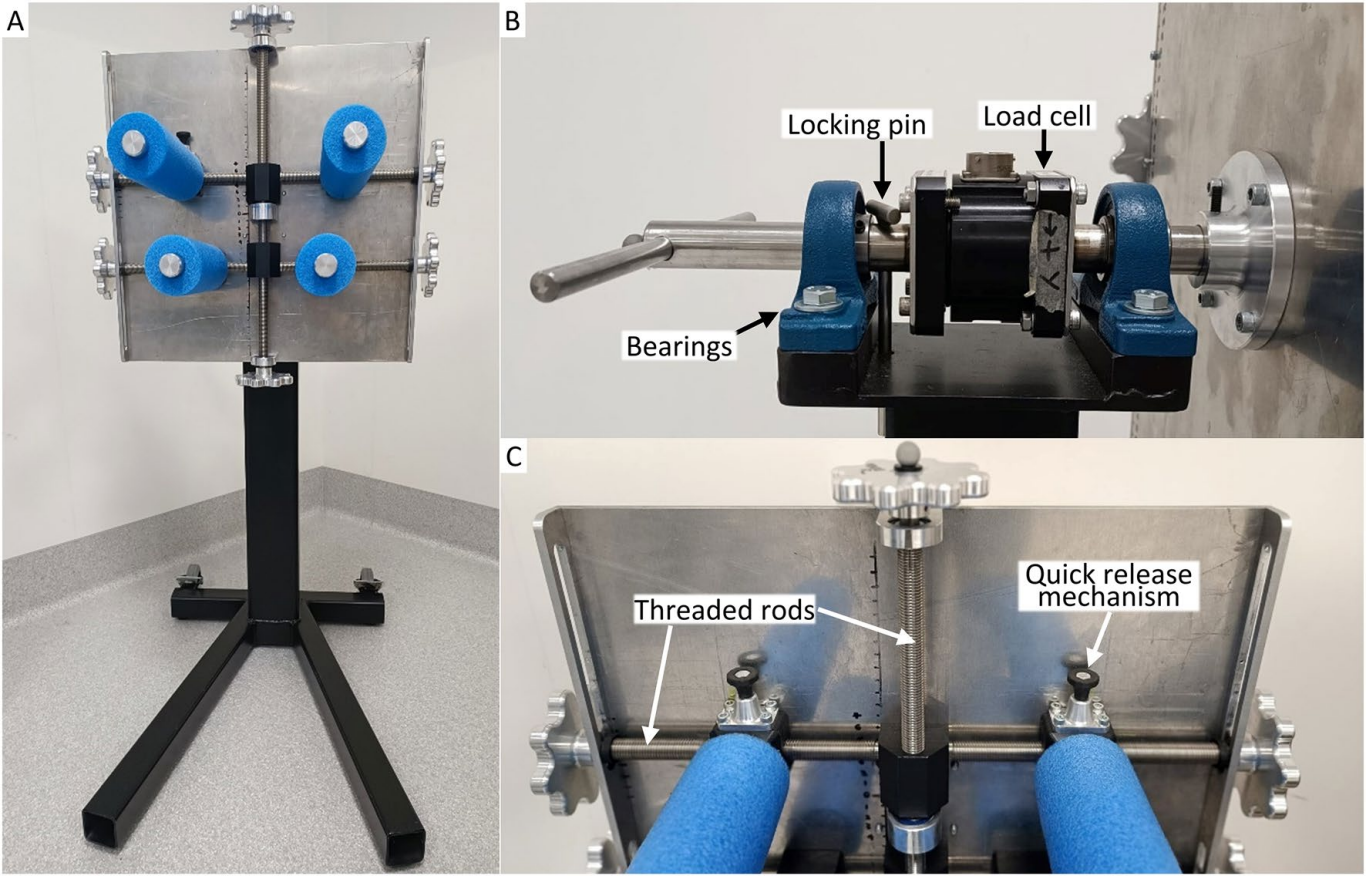
Rotational apparatus. **A** Head plate and stand. **B** Handle, bearings, load cell, and locking pin were linked by the shaft. Locking pin was inserted to the shaft-stand during maximum voluntary contraction tests, to constrain head plate rotation. **C** Position for padded rods can be adjusted along the threaded rods, by turning the knobs on the side of head plate. Quick release mechanisms were installed on top padded rods, to release participant’s head in case of an emergency

The axial rotation apparatus was used to assess axial rotation in the prone position (Fig. 4). The apparatus consisted of a vertical head plate fixed to a horizontal shaft that was supported by two ball bearing units (RS PRO Pillow Block Bearing 25 mm ID, RS Components, Australia) on a vertical stand. Four padded rods were connected to the head plate, and their spacing about the central axis could be adjusted to accommodate differing head geometry. A six-axis load cell (MC3A, AMTI, USA) was mounted on the shaft between the bearings, to measure the axial moment applied to the head-neck. The vertex and superior-inferior axis of the head was visually aligned with the shaft [14]. Reflective markers were placed on the shaft and the stand. The locations of the C4 spinous process, anterior neck surface corresponding to C4, C7 spinous process, and IJ, were recorded in the neutral position to define the location of the neck relative to the apparatus’ fixed axis.

The head plate, load cell (excluding cable), and handle were axis-symmetric about the shaft. However, varying the position of the four padded rods produced asymmetric mass distribution about the apparatus’ axis of rotation. The resulting moment-angle relationship was characterised for each participant-specific head support configuration (without a surrogate head mass), following the participant’s assessment. The mean of five characterisation trials was subtracted from the moment-angle relationship for each of that participant’s axial rotation trials.

### EMG Real-Time Feedback System

A real-time feedback system was used to encourage neck muscle relaxation during “passive” assessments (S.7). EMG signals from the agonist muscles were acquired by the data acquisition unit (Base Station, Delsys Incorporated, USA), then routed to visual and audio paths. In the visual path, following analogue-to-digital conversion of the agonist EMG signals (Lock Lab, Vicon Motion System, UK) the data were processed (full-wave rectified, and root-mean-square smoothed with a 200 ms window) and plotted in real-time (Python 3.7, Python Software Foundation, USA) via Nexus software development kit (Datastream SDK, Vicon Motion System, UK). In the audio path, the agonist voltage signal was amplified by an audio mixer (Zenyx X1222 USB, Behringer, Germany). Then, using a custom unit, the combined agonist EMG signal was amplified by a rotary potentiometer, low-pass filtered (4.7 µF, 470 Ω) and processed by a microprocessor (Arduino Nano, Arduino, USA) to activate a buzzer at a fixed voltage threshold, and adjust the buzzer pitch according to muscle activation. To calibrate the buzzer’s fixed voltage threshold to the participant-specific MVC threshold, the participant performed isometric contraction to 20% MVC, while the signal amplification was adjusted with the rotary potentiometer.

### Data Acquisition and Post-processing

Kinetic, kinematic, and EMG data were synchronised and acquired (Lock Lab, Vicon Motion System, UK), at sample frequencies of 2000 Hz, 100 Hz, and 2000 Hz, respectively. All post-processing was performed in MATLAB (R2020a, MathWorks, USA). Kinetic and kinematic data were filtered with a 4th order bi-directional low-pass Butterworth filter with 10 Hz and 4 Hz − 6 dB cut-off frequencies, respectively. EMG signals were full-wave rectified, then root-mean-square smoothed with a 200-millisecond moving window. After filtering, kinetic and EMG data were down-sampled to 100 Hz, to enable processing with kinematic data.

For each motion direction, the angle of the head relative to the torso in the primary motion direction was described by the primary Euler transformation angle between the defined anatomical coordinate systems. The head rotation was the head-torso angle relative to that of the neutral position, which was recorded immediately after positioning the participant. To assess the consistency with which a similar neutral posture was maintained, the “neutral” head-torso angle was compared for the standing and lying positions. For the bending (flexion, extension, lateral bending) motions, the applied moment was determined by multiplying the applied tangential force, measured by the load cell, by the instantaneous moment arm (IMA). The IMA was defined as the horizontal distance between the load cell reflective marker and the instantaneous centre of rotation (ICR). ICR was calculated in the horizontal plane according to the perpendicular bisector theorem [24, 25], using the location of the two eyelet markers at every 20 frames of the motion capture data. Linear interpolation and linear regression were then used to provide an IMA corresponding to each head-torso angle. The application of non-tangential loads via the cable was evaluated using the forces measured in the two non-primary axes. For passive assessments in which the EMG signal increased throughout the motion arc, passive ROM was defined as the maximum rotational angle prior to the EMG signal exceeding 40% MVC (for either agonist muscle) for 5% of the full ROM (S.8), where the full ROM was defined as the ROM during a passive test regardless of muscle activation. Trials were discarded if passive ROM did not exceed 60% of the full ROM and the active-lying ROM [26].

**Fig. 5:**
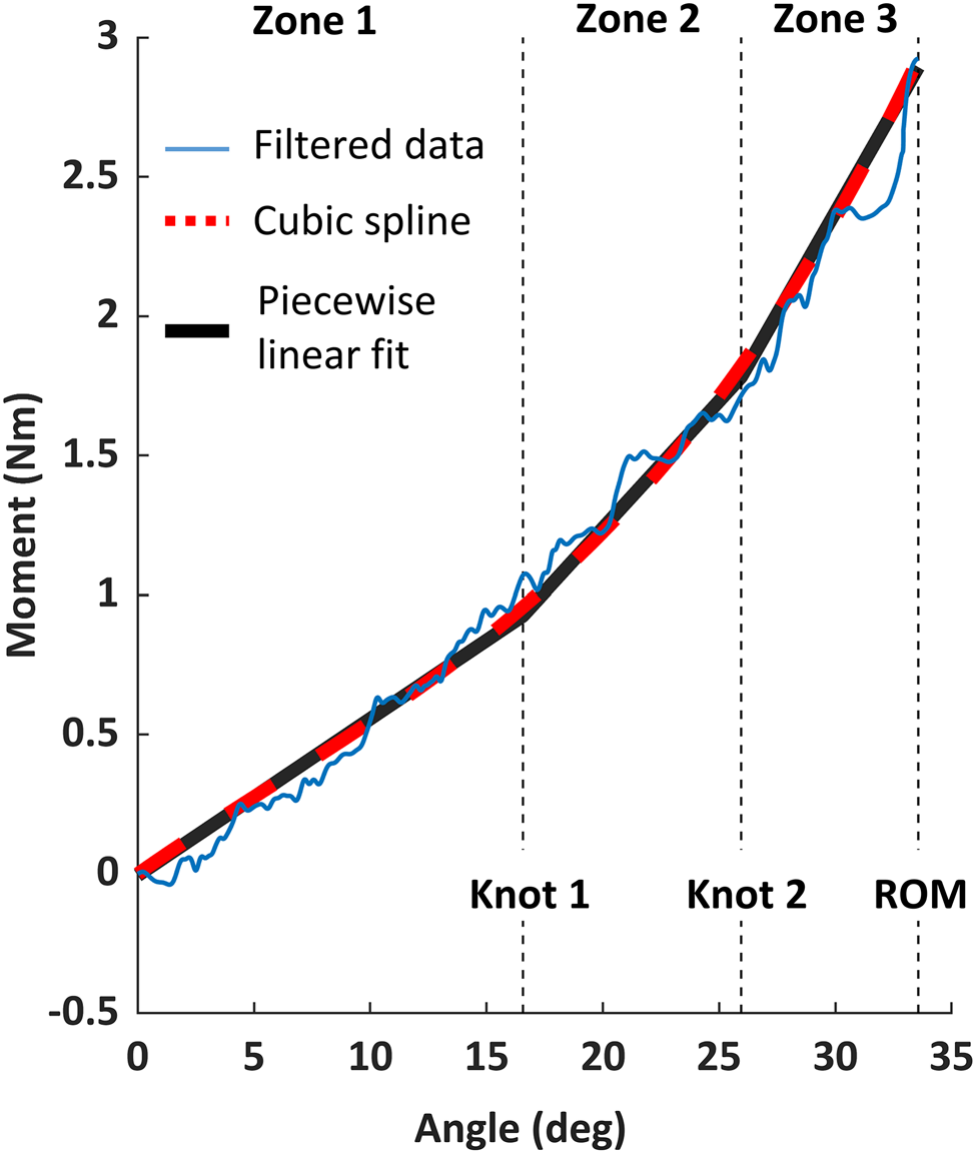
Exemplar moment-angle plot from a left lateral bending trial, showing the filtered data (blue solid line), the cubic spline (red dashed line), and the piecewise linear fit with two trial-specific knots (black solid line). Moment-angle curve starts at zero rotation angle (neutral position)

Because the moment-angle relationship usually appeared to have three linear regions of increasing gradient, neck stiffness was calculated in three zones. For each trial, a cubic spline was fit to the filtered moment-angle data, and then a continuous piecewise linear function with two breakpoints (knots; i.e. at trial-specific angles) [26] was fit to the spline (Fig. 5), using a custom MATLAB program (MATLAB and Shape Language Modelling, MathWorks, USA). The coefficient of determination (*R*^2^) was calculated to assess the goodness-of-fit of the piecewise linear function on the filtered data. Neck stiffness was defined in each zone as the gradient of the corresponding linear function.

### Statistical Analyses

All statistical analyses were performed in SPSS (SPSS 28, IBM, USA). To determine if the repeated measurement caused any fatigue or habituation effects on ROM and stiffness, trial, motion, and session orders were assessed as fixed effects in linear mixed models (LMMs; random effect of participant), for each motion configuration and stiffness zone.

For every motion direction, the between-trial reliability of ROM (active-seated, active-lying, passive-lying) and stiffness in each zone (passive-lying), was assessed by Intraclass Correlation, using a two-way mixed, absolute agreement model, based on averaged measurement. Interclass correlation coefficient (ICC), for session 1 was reported, except for the test configurations for which session 2 had more participants with valid data. The between-session reliability of the mean ROM and stiffness (in each zone), in each motion across all valid trials, was assessed using a two-way mixed, absolute agreement model, based on a single measurement.

To assess the influence of test configuration on the ROM achieved in session 1, the ROM in each motion direction was compared for passive-lying, active-lying, and active-seated configurations, using repeated-measures one-way analyses of variance. Post hoc tests were completed if the between-configuration difference was significant (*p* < 0.05).

## Results

Three male and three female healthy participants (age: 24.8 ± 2.3 years; body mass index: 23.8 ± 2.5 kg/m^2^; head-neck girth ratio: 1.7 ± 0.1) completed two test sessions (14 ± 6 days apart). A total of 1080 trials were recorded and analysed (2 sessions × 6 participants × 6 motions × 3 postures × 5 trials), including 360 passive trials (2 sessions × 6 participants × 6 motions × 1 configuration × 5 trials) for stiffness assessment. Across both sessions, three trials (from three participants) were excluded due to incomplete motion, nine trials (from three participants) were excluded due to occluded head markers and four trials (from four participants) were excluded from bending stiffness assessment due to inconsistent head angular velocity. No fewer than four trials were used to characterise ROM and stiffness for each participant in each test configuration. Moment data for left axial rotation was not recorded for one participant in one test session due to technical difficulties; all statistical comparisons for left axial rotation are for five participants only. For brevity, the reported descriptive statistics for apparatus characterisation, ROM, and stiffness outcomes are for session 1.

### Apparatus, Procedure, and Participant Positioning Assessment

The peak force applied in the non-tangential directions (i.e. caudal-cranial axis, and vertical in the laboratory coordinate system) was 0.5 ± 0.3 N across all flexion, extension, and lateral bending tests (session 1), while the peak force applied in the tangential direction ranged from approximately 4 to 15 N. The mean force associated with friction between the head support and acrylic surface (with surrogate head mass) after applying the surface spray was 2.4 ± 0.6 N, and after each test series this force increased by 0.5 ± 0.4 N, across all participants and configurations. The head moved at 8 ± 2, 27 ± 13, and 34 ± 16°/s, in the passive-lying, active-lying, and active-seated configurations, respectively.

Head and torso coordinate systems were generally aligned in the neutral standing posture, with mean offset of 0 ± 3° about the mediolateral axis, 0 ± 3° about the anterior-posterior axis, and 1 ± 4° in the caudal-cranial axis. Across all lying configurations, the neutral position corresponded to a 2 ± 9° extension, 7 ± 7° left lateral rotation, and 3 ± 6° right axial rotation, compared to the neutral standing posture. The between-session difference for these offset angles was 7 ± 6°, 3 ± 3°, 5 ± 3°, respectively (S.9). In the axial rotation configuration, the apparatus axis passed through the anterior-posterior bounds of the participant’s neck at the C4 and C7 level (S.10).

Trial number had no effect on ROM (S.11), except for increased passive-lying (*p* = 0.002) and active-seated (*p* = 0.019) ROM in left axial rotation and active-seated ROM in right axial rotation (*p* < 0.001). Stiffness in flexion zone 3 (*p* = 0.041) and left axial rotation zone 2 (*p* = 0.038) increased with trial number, while other zones showed no association. The effect of motion order on ROM had little consistency across motions and configurations (S.11). Stiffness in zone 2 of left lateral bending (*p* = 0.027), zone 1 (*p* = 0.032) and 2 (*p* = 0.016) of right lateral bending, and zone 1 of left axial rotation (*p* = 0.002) decreased with motion order, but no association was found for the other stiffness zones.

### Range of Motion

**Fig. 6:**
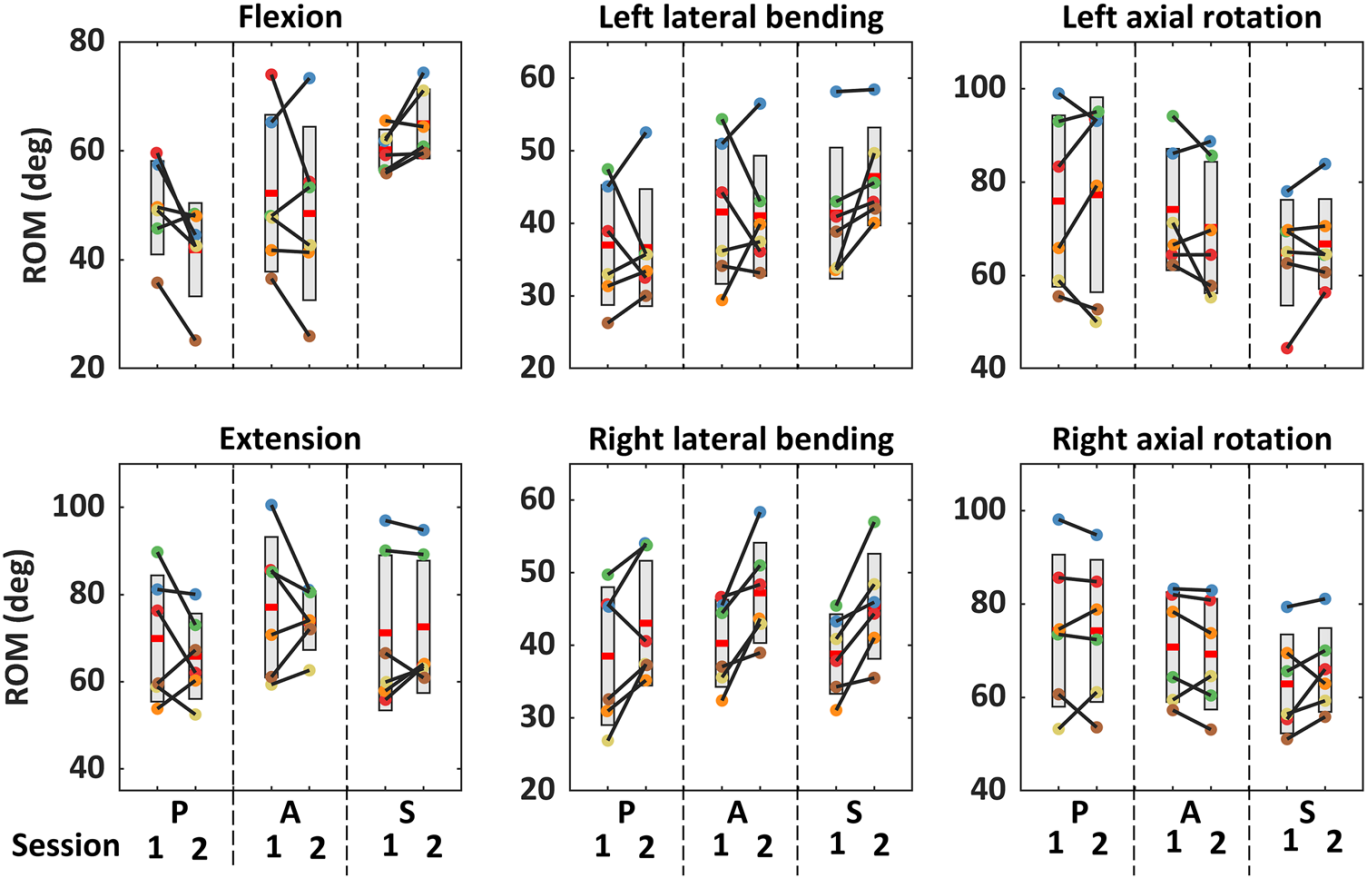
Head-neck range of motion (ROM; *P* passive-lying, *A* active-lying, *S* active-seated). Box is mean ± standard deviation for all participants. Each colour represents one participant. Each dot is the mean ROM across all trials for the same configuration, for each session

No passive trials were excluded due to high muscle activation or insufficient motion; passive ROM exceeded 60% of full ROM and active-lying ROM for all trials (S.12). Passive-lying, active-lying, and active-seated ROM were similar, except in flexion for which active-seated ROM was significantly greater than passive-lying ROM (Table 2). Across all test configurations, participants with lower ROM in session 1 generally had lower ROM in session 2 (Fig. 6).

**Table 2:** Range of motion in degrees (mean ± standard deviation) for each test configuration and motion direction (session 1), and the outcome of the repeated-measures one-way analysis of variance comparison between configurations

	Flexion	Extension	Left lateral bending	Right lateral bending	Left axial rotation	Right axial rotation
Passive lying	50 ± 9	70 ± 14	37 ± 8	39 ± 9	75 ± 17	74 ± 15
Active lying	52 ± 14	77 ± 16	42 ± 10	40 ± 6	74 ± 13	71 ± 12
Active seated	60 ± 4	71 ± 18	41 ± 9	39 ± 5	65 ± 11	63 ± 11
Between-configuration *p*	0.006*	0.358	0.106	0.498	0.276	0.214

*Post hoc analysis outcomes. Passive-lying versus active-lying: *p* = 0.436. Passive-lying versus active-seated: *p* = 0.018. Active-lying versus active-seated: *p* = 0.239

Between-trial agreement for ROM was mostly excellent across all assessment configurations (Table 3). Between-session agreement for ROM was moderate-to-excellent for most configurations. Agreement was poor in flexion (passive-lying, active-seated), and right lateral bending (active-lying, Table 4).

**Table 3:**
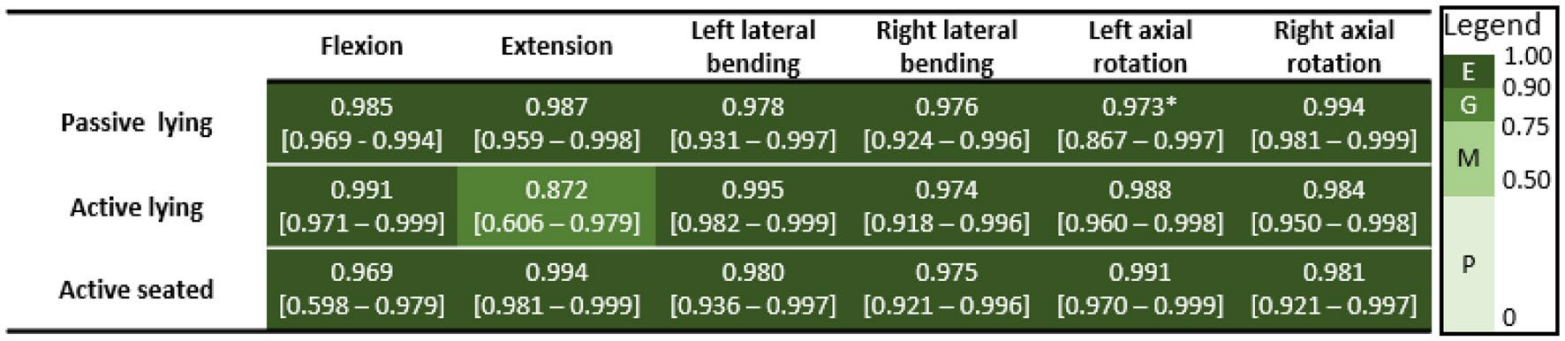
Between-trial intraclass correlation coefficient (ICC) [95% confidence interval] for head-neck range of motion in each test configuration and each motion direction, with ICC rating mapped to colour according to the provided legend (*E* excellent, *G* good, *M* moderate, *P* poor)

*From 5 participants that had 5 valid trials

**Table 4:**
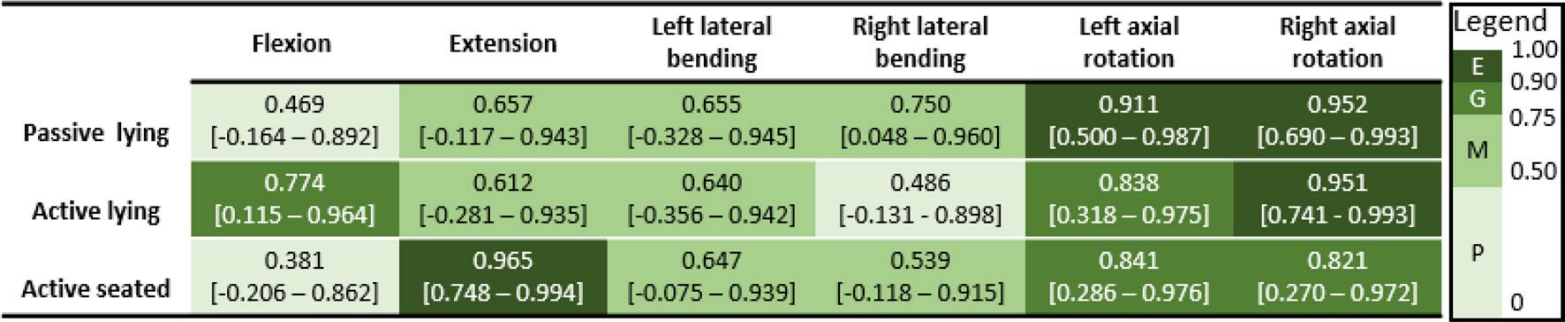
Between-session intraclass correlation coefficient (ICC) [95% confidence interval] for head-neck range of motion in each test configuration and each motion direction, with ICC rating mapped to colour according to the provided legend (*E* excellent, *G* good, *M* moderate, *P* poor)

### Stiffness

**Fig. 7:**
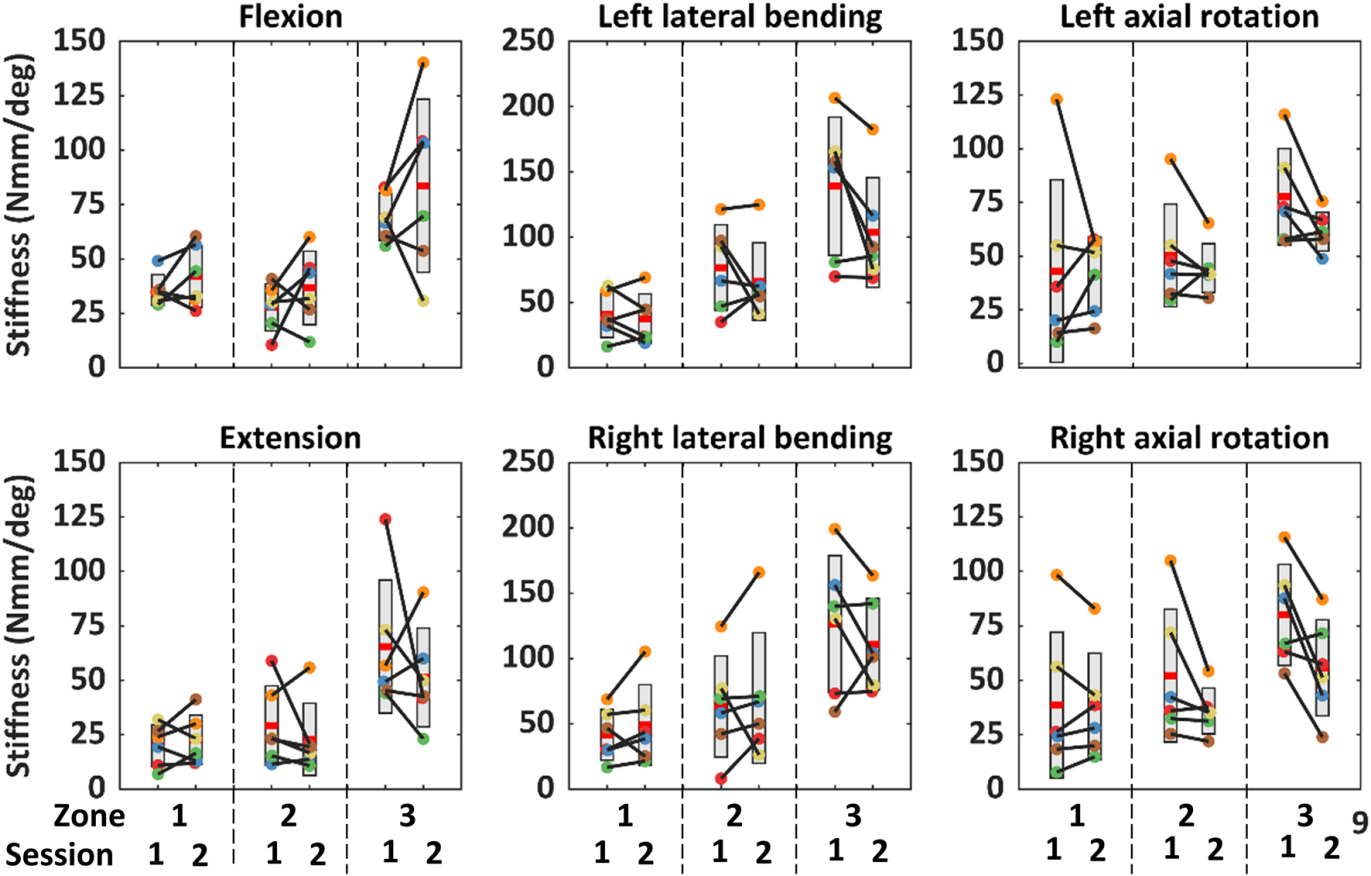
Passive lying head-neck stiffness. Box is mean ± standard deviation for all participants. Each colour represents one participant. Each dot is the mean stiffness across all trials for the same configuration, for each session

Stiffness generally increased across the three zones (Table 5, S.13). The piecewise continuous linear function approximated the moment-angle filtered data, with *R*^2^ of 0.961 ± 0.019 across all assessment configurations. Flexion stiffness in session 2 were up to 32% (zone 2) lower than session 1 (Fig. 7), whereas zone 2 and 3 extension stiffness were 22% lower. Between-session stiffness difference for lateral bending was within 20% of session 1, except left zone 3 stiffness in session 2 had 25% increase. Right axial rotation generally had larger between-session difference than left axial rotation.

**Table 5:** Head-neck stiffness in each zone in each motion direction, in the passive-lying configuration, from session 1 (Unit: Nmm/deg)

	Flexion	Extension	Left lateral bending	Right lateral bending	Left axial rotation	Right axial rotation
Zone 1	36 ± 7	20 ± 10	41 ± 17	42 ± 19	43 ± 43	39 ± 33
Zone 2	28 ± 11	29 ± 18	77 ± 33	63 ± 39	50 ± 24	52 ± 30
Zone 3	69 ± 11	65 ± 31	139 ± 53	126 ± 52	78 ± 22	80 ± 23

Between-trial agreement for stiffness was good-to-excellent in lateral bending and axial rotation, and moderate-to-good in flexion and extension (Table 6). Between-session agreement for stiffness in lateral bending was generally moderate-to-good, and in flexion and axial rotation in some zones was poor (Table 7).

**Table 6:**
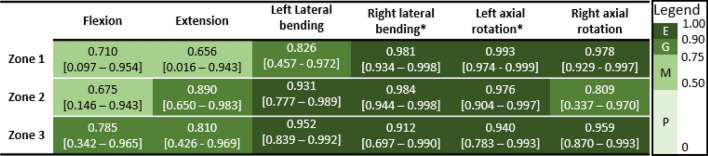
Between-trial intraclass correlation coefficient (ICC) [95% confidence interval] for head-neck stiffness in each test configuration and each motion direction, with ICC rating mapped to colour according to the provided legend (*E* excellent, *G* good, *M* moderate, *P* poor)

*From 5 participants that had 5 valid trials

**Table 7:**
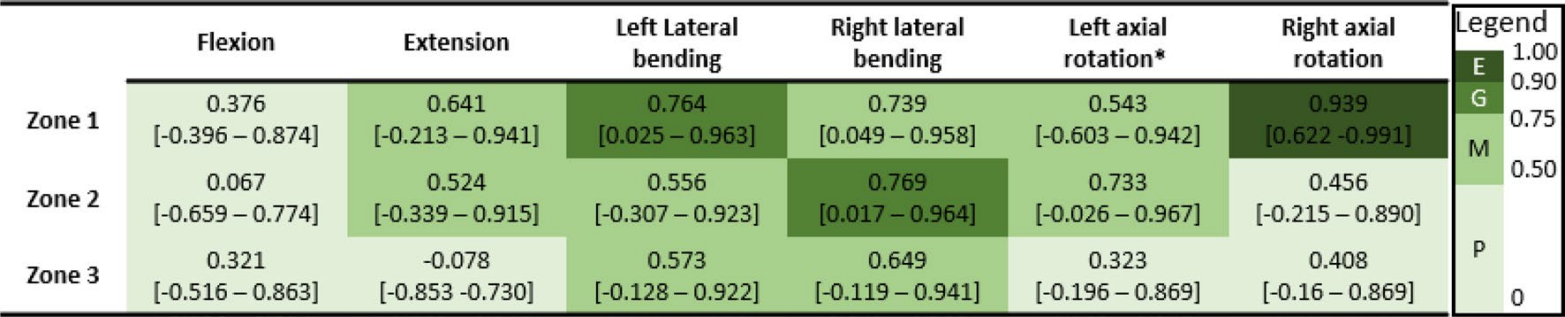
Between-session intraclass correlation coefficient (ICC) [95% confidence interval] for head-neck stiffness in each test configuration and each motion direction, with ICC rating mapped to colour according to the provided legend (*E* excellent, *G* good, *M* moderate, *P* poor)

*From 5 valid participants (1 participant did not have moment data)

## Discussion

Well-defined passive stiffness and ROM, about each anatomical axis, for the healthy human neck is important for a variety of clinical and bioengineering applications. This study describes the development and assessment of apparatus and protocols to measure passive neck stiffness and ROM in the lying position. Participant head-torso alignment, bending apparatus loads due to friction, and non-tangential pulling direction, were evaluated. Passive stiffness and ROM were assessed for six young healthy participants in two repeated sessions. ROM was generally similar for different configurations, and stiffness generally increased with zones. Between-session reliability was low in some motions, but the between-trial reliability was mostly good-to-excellent.

Our bending and axial rotation apparatus were nominally based on the designs of McGill et al. [16], and Dugailly et al. [14], respectively. During apparatus design and commissioning, we identified several potential sources of variation, which led to specific design features, and protocols for apparatus operation and participant-apparatus interaction. For the bending apparatus, the use of eyelets to guide the direction of the applied load resulted in off-axis loads less than approximately 10% of the load in the tangential (primary) direction. The load due to friction between the head support and the acrylic surface (with a surrogate head) was nominally 15% to 50% of the peak load applied during participant testing. It was not feasible to correct the moment-angle test data for this intrinsic apparatus load in a participant-specific manner, rather it was monitored. McGill et al. [16] did not report a friction load for their similar apparatus. These data suggest that for this apparatus and protocol, the derived neck stiffness values indicate an upper bound, particularly for the low stiffness observed in Zone 1. For the axial rotation assessment, although the effect of participant-specific non-axis-symmetric head support mass distribution was accounted for, the effect of head and neck mass distribution about the shaft could not be decoupled from the effect of neck stiffness. When mounted in the axial rotational apparatus, the axis of rotation of the shaft passed through vertex of the participants’ head and was within the boundary of the neck (S.10), ensuring that this effect was minimised.

For both bending and axial rotation apparatus, neutral head-torso alignment could affect ROM [27], and likely stiffness, measurements. It was challenging for participants to self-identify the neutral posture in prone and side-lying positions with the head and torso fixed to independent surfaces. Recording the standing neutral sagittal alignment with the flexible contour ruler provided a target position to achieve in the lying positions. Although attempted for the coronal plane, it was not successful due to the dissimilar shoulder position in side-lying; consequently, coronal and transverse alignment was by visual inspection. Across all assessment configurations, the offset in head-torso angle between standing neutral and the lying positions was generally lower than 17° across all axes, and the between-session difference in offset was generally less than 14°. In addition, head-neck placement in the rotational apparatus was reasonably repeatable (S.10), and these data suggested that between-subject variation in head-torso alignment was greater than the variation imposed by the apparatus or protocol.

We assessed active-seated and active-lying ROM, in an effort to confirm that the apparatus’ and/or researcher-initiated motion did not substantially limit the participants’ ability or willingness to achieve a “true” passive-lying ROM. For these participants, there were no systematic differences in the ROM achieved across the three postures, except for the flexion motion in which active-seated ROM was greater than passive-lying ROM. There was no difference between active-lying and passive-lying ROM in flexion. Flexion is the only passive-lying configuration in which the participant has direct view of the researcher, the cable, and the proximity of their head to the edge of the acrylic surface. This may have contributed to the lower ROM achieved in this configuration.

The passive-lying neck stiffness and ROM measured in this study were similar to those previously reported. In participants of similar age, McGill et al. [16] reported passive-lying neck stiffness in flexion, extension, and lateral bending, by taking the derivative (at 10° intervals) of an exponential function fit to the moment-angle data. Stiffness for female participants overlapped with the current study, but stiffness for males and closer to end-of-ROM was higher than this study (S.14). Force associated with apparatus surface friction, participant alignment procedures, and muscle activity protocols were not reported in McGill et al. [16]; such factors may have contributed to the difference in measured stiffness’. Dugailly et al. [14] assessed passive-lying neck stiffness and ROM in axial rotation for an older cohort (48 ± 14 years), defining stiffness as the moment-angle gradient in the last 10 degrees of motion [1]. The reported ROM (Left: 83 ± 16°, Right: 74 ± 15°) was similar to the current study (Left: 76 ± 18°, Right: 74 ± 16°), but the mean stiffness (Left: 84 ± 31 Nmm/deg; Right: 89 ± 35 Nmm/deg) was slightly higher than the current study (Zone 3; Left: 78 ± 22 Nmm/deg, from 58°; Right: 80 ± 23 Nmm/deg, from 57°; S.15). In that study, muscle activation was not monitored, and if present it could have contributed to the higher stiffness observed [28]. The study does not report compensating for any artifactitious apparatus moment throughout rotation; this could affect stiffness calculations if the apparatus moment varied throughout ROM.

Although the similarity of moment-angle and stiffness data across trials could be qualitatively assessed graphically, stiffness in three zones was used to perform quantitative ICC analyses. Calculating stiffness in three regions was inspired by a study of lumbar spine stiffness conducted with a similar bending apparatus [29]. The “knots” between each stiffness zone did not necessarily have physiological meaning, instead they mathematically represented the dominant locations of gradient change on the moment-angle plot. For lumbar spine moment-angle curves, Barrett et al. [26] reported that the piecewise curve fitting method was reliable if the assessed region covered more than 60% ROM. In the current study, all passive ROM exceeded 60% full ROM and 60% active-lying ROM.

ICR was primarily used for moment arm calculation to determine stiffness for passive tests; however, it was also compared to the ICR for active lying to qualitatively evaluate the similarity in head motion path (relative to the torso) between these two test configurations. In the passive-lying configuration, the ICR was located around C7 for lateral bending, around the mid-cervical region for flexion, and around the anterior mid-length for extension (S.16). For active-lying configurations, the ICR was generally located in similar regions for lateral bending and extension, but variation was observed throughout the motion and between sessions. These data suggest the head rotated in broadly similar paths in these passive and active configurations, but potentially with less consistency when self-controlled. There was markedly greater variation in ICR location in active flexion. Some of the ICR change observed from neutral to maximum ROM could be due to the use of the perpendicular bisector theorem which is susceptible to inconsistent motion between adjacent calculation steps due to self-determined head movement speed. Kuo et al. [30] assessed ICR during frontal and lateral impacts by releasing a weight that attached to the head in a seated position, and their extension ICR was in a similar region to ours, but their lateral bending ICR was inferior to C7. In active-seated flexion and extension, Lee et al. [25] reported ICR was mostly inferior and posterior to T1. Despite changes in ICR location, increased moment throughout range of motion was more substantially due to the applied force (which increased from 1-3 N to 5-13 N) than the IMA (0.35–0.5 m). In the axial rotation configuration, the location and orientation of the rotational axis may affect the head-neck stiffness. The estimated rotational axis in passive-lying was similar to that in active-lying, but differed from that in the active-seated configurations for some participants (S.17), potentially due to head-torso postural difference.

The between-trial reliability of ROM was excellent for every test configuration (ICC > 0.969) except extension in active-lying (ICC = 0.872). The confidence intervals were narrow (generally less than 0.1), providing confidence in the estimated ICCs. The between-trial reliability of neck stiffness was good-to-excellent across most of the test configurations and zones (ICC > 0.785), but was moderate in the lower stiffness zones in flexion and extension (ICC: 0.656–0.710). These lower ICCs had relatively broad confidence intervals, suggesting these estimates may lack accuracy. Tests performed on the rotational apparatus generally had higher between-trial ICC than those performed on the bending apparatus. This may be because the rotational apparatus could only affect motion in one rotational degree of freedom, whereas the head-neck had two (translational) degrees of freedom in the bending apparatus. Overall, this reliability assessment indicated that for a given participant-apparatus interaction and posture, and in the absence of day-to-day intrinsic variabilities in the participant, researcher or environment, the outcomes have acceptable repeatability.

The between-session reliability of ROM and stiffness was generally lower than between-trial reliability. In addition, the wide confidence intervals of these ICCs suggested more participants might be required to confirm the reliability results. The lower reliability and the variation in ICC across the test configurations were likely reflective of factors including: intrinsic physiological participant variance, inconsistencies in participant-apparatus interaction (particularly head-neck-torso posture) that effect head-neck mechanics, and unintentional differences in implementation of protocols. The low ICCs may indicate that these apparatus and protocols should be assessed more carefully before using them to evaluate participant-specific changes in neck mechanics resulting from interventions or similar.

**Fig. 8:**
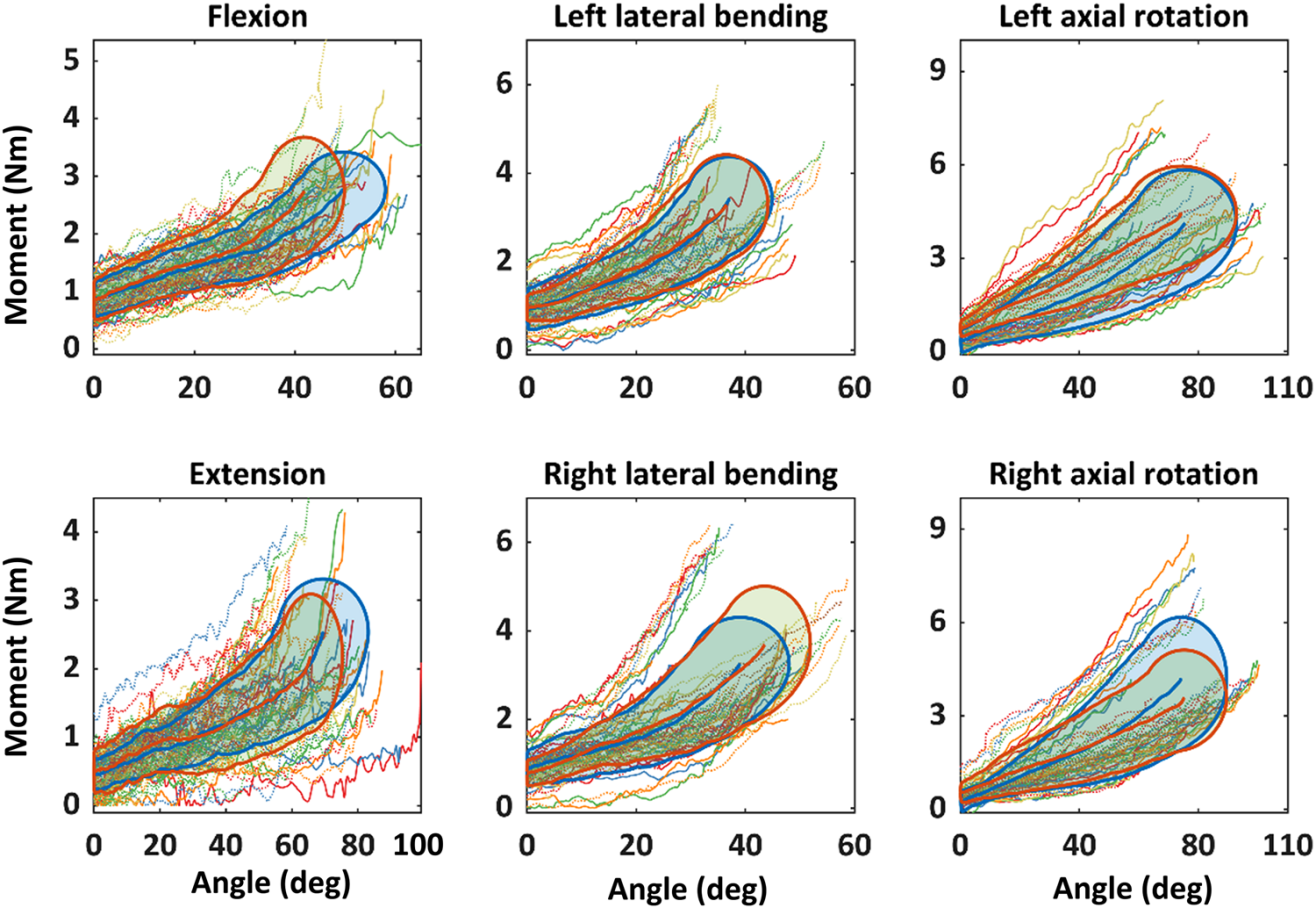
Moment-angle curves from all trials in both sessions. Each corridor corresponds to moment-angle data that were within one standard deviation from the mean, generated by an automated arc-length re-parametrization method. Bold lines represent the corridor boundary and the mean moment-angle curve corresponding to session 1 (blue) and session 2 (red). Solid and dashed thin lines represent the moment-angle plots from each trial in sessions 1 and 2, respectively, for which each colour corresponds to one participant

A potential use of these protocols is to develop normative reference limits (i.e. corridors) for ROM and stiffness for specific populations. We attempted to assess the extent to which the stiffness, ROM, and moment-angle corridors were similar across the two sessions, at a cohort level, despite the relatively low between-session ICCs. Stiffness corridors were developed via arc-length re-parametrisation method [31], which suggested the moment-angle curves were most similar for extension and lateral bending, but less similar in axial rotation and flexion towards end of ROM (Fig. 8). This trend was also observed for the stiffness corridors developed from mean stiffness values of each participant (S.18).

There were several limitations in this study. Limits of ROM were participant-guided in both the active and passive tests; while this ensured participant safety, it may have contributed to variability of ROM and stiffness zones between sessions. Participants had open or closed eyes during lying tests, a preference which provided comfort and potentially assisted relaxation, but those with eyes open may have shortened their ROM due to the view of researcher and apparatus (particularly in flexion). Passive human neck stiffness is low, thus any inconsistency in intrinsic and extrinsic factors related to the participant, apparatus, and participant-apparatus interaction (e.g. pulling speed, surface friction, marker placement, participant alignment) could reduce repeatability, despite adherence to test protocols. Moreover, although all participants reached 60% full ROM and active-lying ROM, it is possible that some of the variability in zone 3 was due to different passive ROM being achieved on the two test days.

The term ‘MVC’ in this study refers to the muscle contraction resulting from producing maximal effort, with the head-neck in the neutral position, against constraints in each test direction. This maximal effort was the result of all contributing neck muscles, and did not provide an MVC, and the associated activation level, for individual muscles. Although participants were verbally encouraged by the researcher to produce maximal effort during the MVC data collection, it is possible that maximum effort was not achieved. The combination of these two factors in certain cases yielded high normalised EMG values that would generally not be considered physiological; caution should be taking interpreting these values as directly relating to single-muscle exertion. Additionally, the location of the electrodes relative to the muscle fibres (at neutral) may have changed throughout the large ranges of motion tested. Placement and alignment of the electrodes were expected to yield crosstalk from trapezius and levator scapulae muscles into the signal detected by the SPL electrodes [32]. Although a personalised, neck angle-specific, and muscle-specific, contraction threshold may have better confirmed the passive condition through the range of motion, a standard constant threshold based on the neutral posture was more practical in this extensive testing protocol.

A within-day repeated test could assess apparatus and protocol reliability with potentially reduced physiological effects, but was not conducted due to logistical constraints. However, the between-session repeated test provided an overall understanding of the test reliability. The number of participants was relatively low, although it was similar to Dugailly et al. [14], but less than McClure et al. [15]. Confidence in the ICC estimates for the between-session reliability analysis, and the apparent effects of trial and motion order on ROM and stiffness in some configurations, may be enhanced by evaluating more participants.

In conclusion, this study evaluated apparatus and protocols to characterise passive head-neck ROM and stiffness in flexion, extension, lateral bending, and axial rotation, in the lying position. Between-trial reliability was acceptable across all motions, but between-session reliability was poor in flexion, and in some stiffness zones in extension and axial rotation, despite the protocol achieving relatively consistent neutral postures. Although confidence in the ICC outcomes may be limited due to wide confidence intervals, the relatively poor between-session reliability indicates that this assessment may not be suitable to detect subtle changes in multi-session repeated-measures or longitudinal studies. However, the presented methods are sufficiently robust to characterise neck stiffness and ROM across population samples of suitable size where inter-individual variability is expected.

### Supplementary Information

Below is the link to the electronic supplementary material.

Supplementary file1 (PDF 2627 KB)

## Data Availability

The data that support the findings of this study can be found in Figshare repository (10.25909/c.7016496).

## Abbreviations

ROM: Range of motion
CROM: Cervical range of motion
L_T: Left tragion
R_T: Right tragion
L_IO: Left orbit inferior margin
R_IO: Right orbit inferior margin
L_MP: Left mastoid process
IJ: Sternal notch
XP: Xiphoid process
EMG: Electromyography
SCM: Sternocleidomastoid
SPL: Splenius muscle
TRP: Trapezius muscle
MVC: Maximum voluntary contraction
ICC: Intraclass correlation coefficient
IMA: Instantaneous moment arm
