# Supplementary material for: Evaluation of Apparatus and Protocols to Measure Human Passive Neck Stiffness and Range of Motion
Source: Ann Biomed Eng. 2024 Apr 24;52(8):2178–92. doi: 10.1007/s10439-024-03517-w (PMC11247060; doi:10.1007/s10439-024-03517-w)
Supplement: Supplementary file 1 — Supplementary file1 (PDF 2627 KB) [file 10439_2024_3517_MOESM1_ESM.pdf]

SUPPLEMENTARY MATERIALS

S.1 Marker and EMG placement

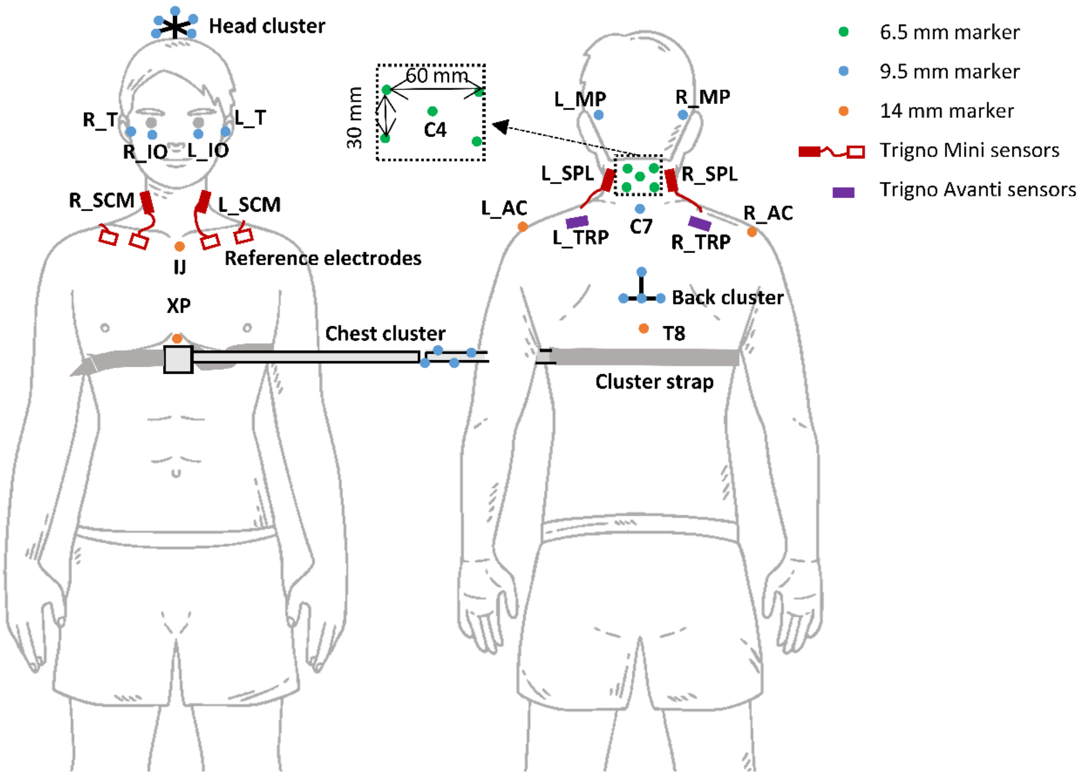

Figure S.1: Schematic illustrating reflective marker and electrode placement. L_T and R_T: left and right tragion. L_MP: left mastoid process. L_IO and R_IO: left and right orbit inferior margin. L_SCM and R_SCM: left and right sternocleidomastoid muscles. L_SPL and R_SPL: left and right splenius muscles. L_TRP and R_TRP: left and right trapezius muscles. IJ: sternal notch. XP: xiphoid process. Additional markers were placed on the left and right acromion (L_AC and R_AC), right mastoid process (R_MP) and C4 regions, but were not used in the analysis.

S.2 Head and torso coordinate system

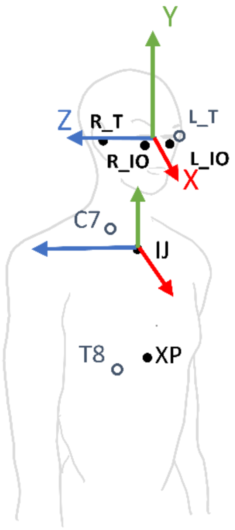

Figure S.2: Head and torso coordinate system. L_T and R_T: left and right tragion. L_IO and R_IO: left and right orbit inferior margins. IJ: sternal notch. XP: xiphoid process. Head +Z: passing through L_T and R_T, pointing to the right. Head +X: normal to Z-axis, passing through mid-point of L_IO and R_IO, pointing anteriorly. Head +Y: orthogonal to X and Z axis, pointing superiorly. Torso +Y: passing through XP-T8 and IJ-C7 mid-point, pointing superiorly. Torso +Z: orthogonal to the plane formed by IJ, C7 and mid-point of XP-T8, pointing to the right. Torso +X: orthogonal to Y and Z axis, pointing anteriorly.

S.3 Neutral alignment with flexible ruler

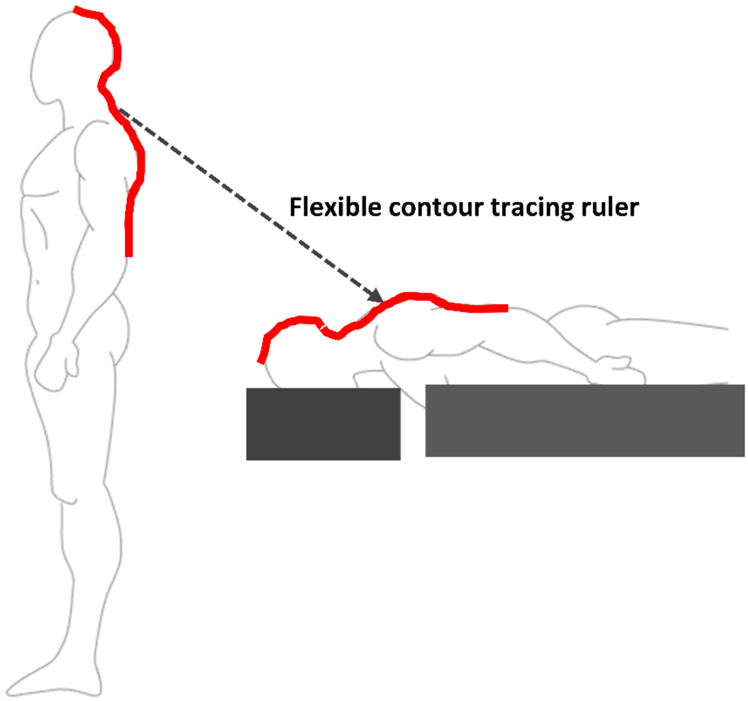

Figure S.3: Schematic diagram of using flexible contour tracing ruler in prone position. A flexible contour-tracing ruler was used to maintain the participants' standing neutral head-neck-torso alignment, when in the prone and side-lying position. In the standing position, the flexible ruler was contoured to the participants' head, neck and upper torso. Then, the participant lay in the prone position, the pre-contoured ruler was replaced, and the apparatus was adjusted until the participant's head, neck and upper torso closely matched the contour of the ruler.

S.4 Maximum voluntary contraction

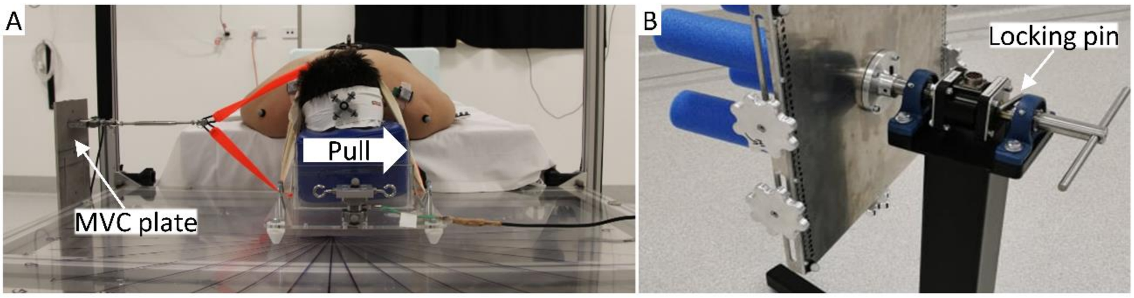

Figure S.4: Maximum voluntary contraction (MVC) test performed at neutral position (0°). (A) Lateral bending configuration: the head and head support were fixed to a rigid aluminium plate via a cable. Plate height and position were adjusted to align the cable with the centre of the head support and the head. (B) Axial rotation configuration: a locking pin was inserted through the shaft into the stand to eliminate shaft and head rotation.

S.5 EMG and passive threshold

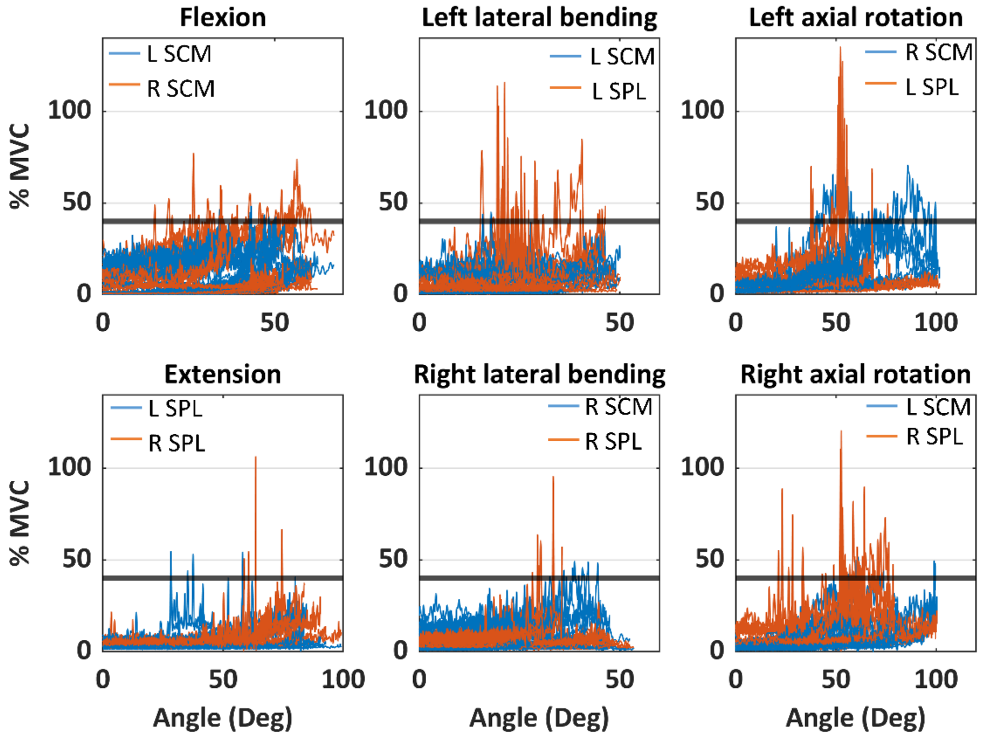

Figure S.5.1: Electromyography (EMG) signals for agonist muscles in each lying-passive motion, for all participants and all trials, normalised to the EMG from maximum voluntary contraction (MVC) test performed at neutral position (nominally 0°). Black horizontal lines represent the passive EMG threshold at 40% MVC. L SCM and R SCM: left and right sternocleidomastoid muscles. L SPL and R SPL: left and right splenius muscles. L TRP and R TRP: left and right trapezius muscles.

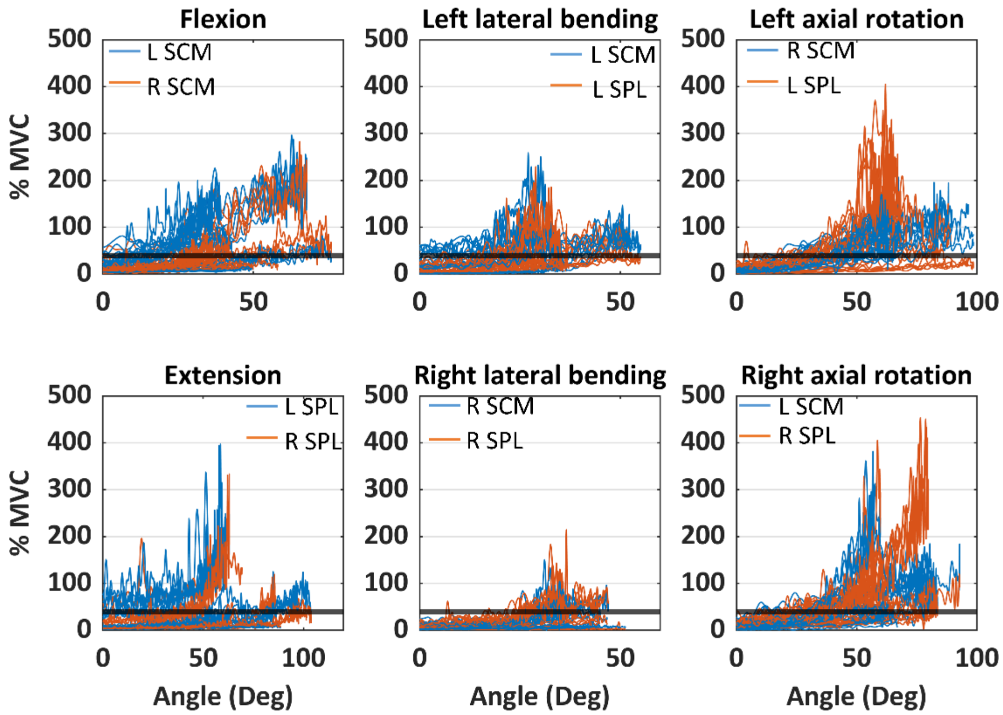

Figure S.5.2: Electromyography (EMG) signals for agonist muscles in each lying-active motion, for all participants and all trials, normalised to the EMG from maximum voluntary contraction (MVC) test performed at neutral position (nominally 0°). Black horizontal lines represent the passive EMG threshold at 40% MVC. L SCM and R SCM: left and right sternocleidomastoid muscles. L SPL and R SPL: left and right splenius muscles. L TRP and R TRP: left and right trapezius muscles.

S.6 Bending apparatus, force due to friction between head support and acrylic surface, with surrogate head mass.

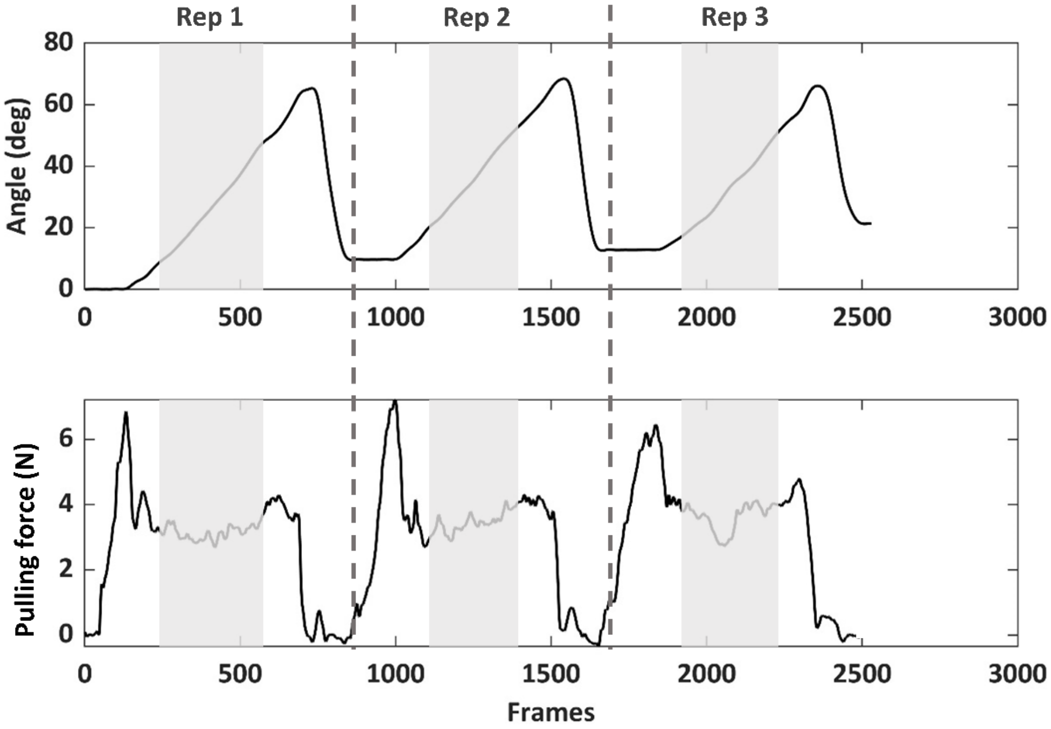

Figure S.6: (A) Head support angle and (B) tangential pulling force due to friction, from one friction test with three consecutive repetitions (dotted lines). Surface friction was the mean tangential pulling force from three plateau regions (shaded in grey). Repetition (rep) 2 and 3 did not start at the initial position.

S.7 EMG feedback system

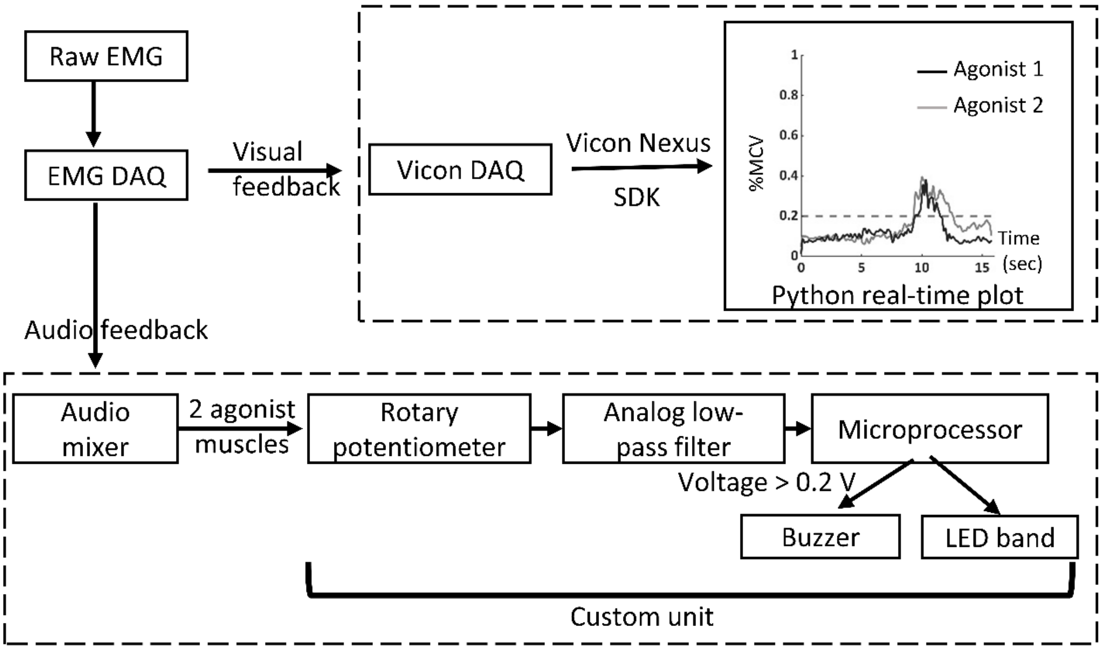

Figure S.7: Electromyography (EMG) real-time feedback system (DAQ: data acquisition system, SDK: software development kit). To aid calibration of the buzzer's voltage threshold to the muscle activation threshold, a band of emitting diodes (LEDs) was illuminated to visually indicate the microprocessor output voltage level.

S.8 EMG and passive ROM

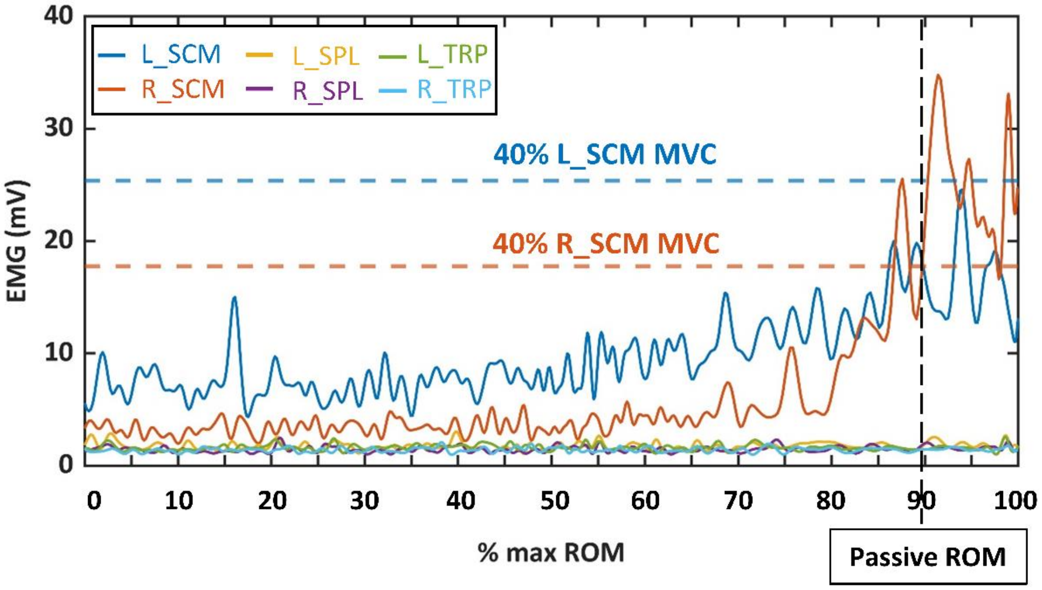

Figure S.8: Exemplar electromyography (EMG) versus percentage of maximum range of motion (ROM), for a passive-lying flexion trial. L_SCM and R_SCM were the agonist muscles. Passive range of motion (ROM) was defined as the motion prior to the muscle activity exceeding the 40% MVC threshold for 5% ROM (black dashed line). L: left, R: right, SCM: sternocleidomastoid muscle, SPL: splenius muscle, TRP: trapezius muscle.

S.9 Head-torso alignment in lying position

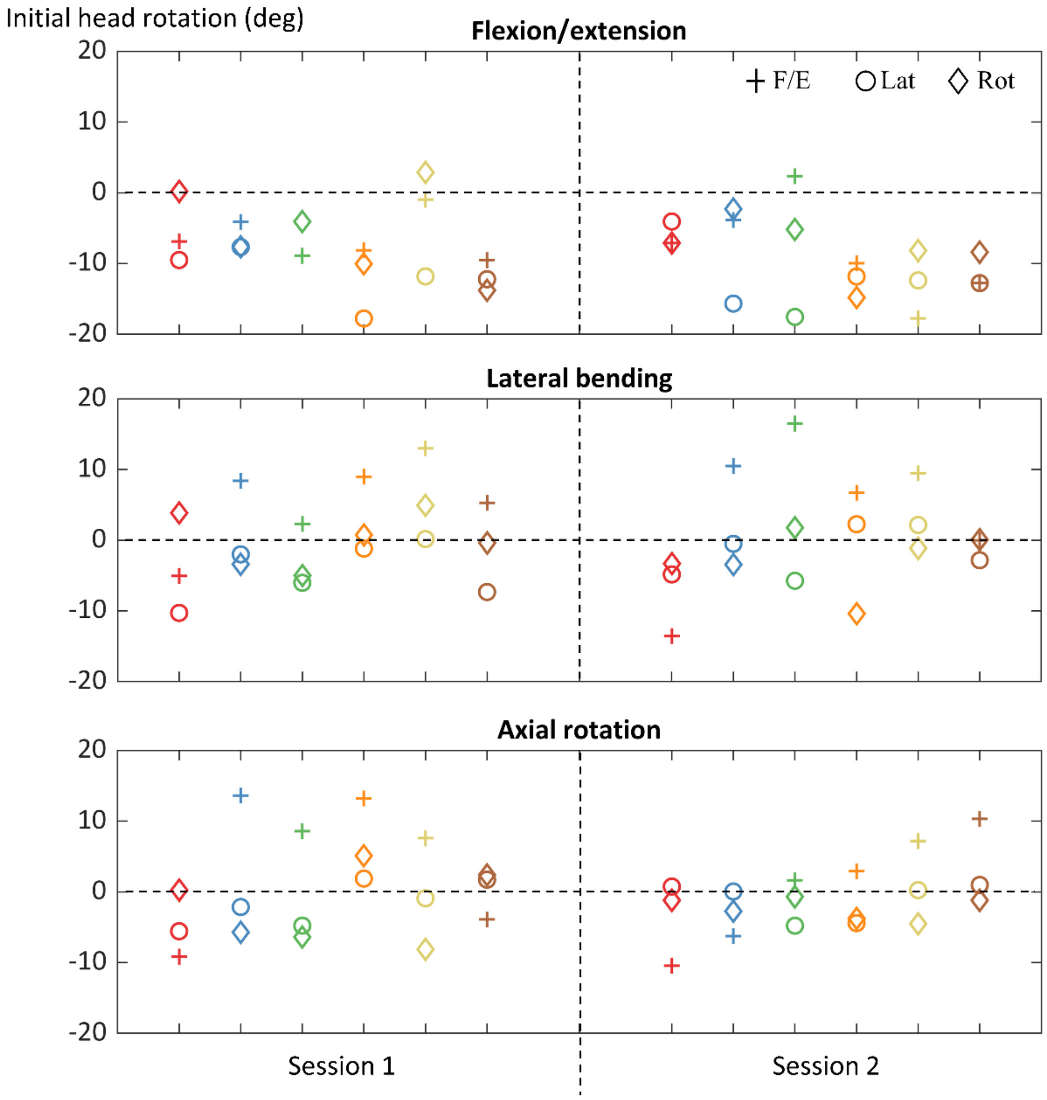

Figure S.9: Head-torso angle, relative to the neutral standing position, about flexion/extension axis (F/E; flexion: negative, extension: positive), lateral bending axis (Lat; left: negative, right: positive) and axial rotation axis (Rot; left: positive; right: negative) immediately prior to flexion/extension, lateral bending and axial rotation tests, for each participant in both sessions. Participant colour is consistent with rest of the manuscript.

S.10 Neck marker vertical position relative to the apparatus centre of rotation.

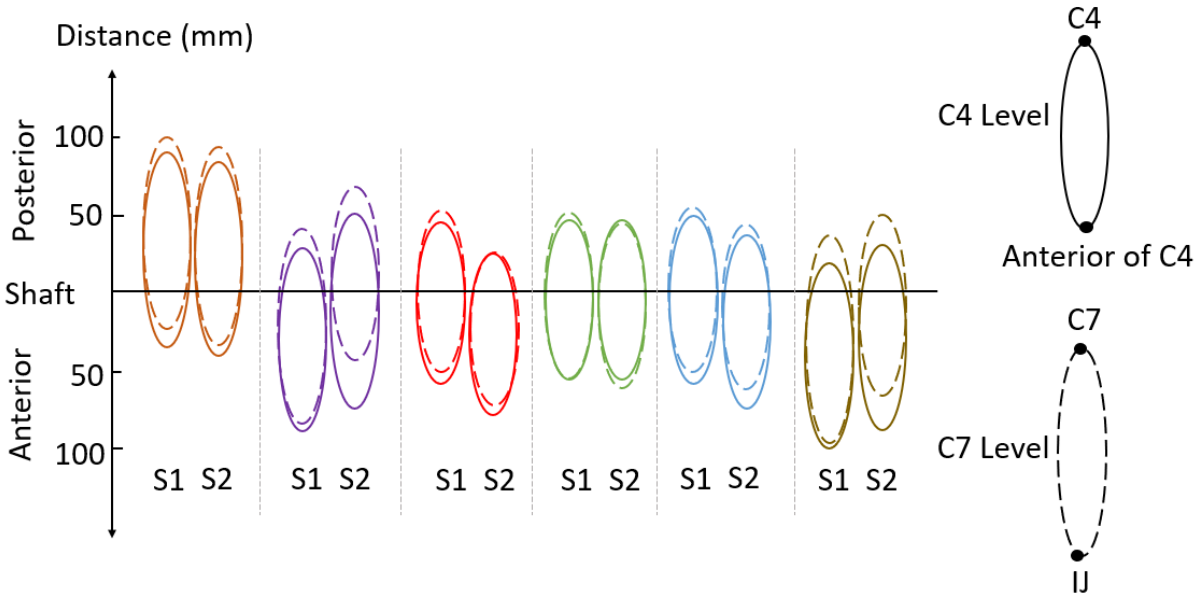

Figure S.10: Neck position in the neutral posture immediately prior to axial rotation motion test commencement, relative to the apparatus shaft, for each participant (indicated by colour), for session 1 (S1) and session 2 (S2). The ellipse major dimension corresponds to the vertical distance between C4 and the anterior neck position at C4 (solid line), and the vertical distance between C7 and the sternal notch (IJ) (dashed line). The centre of rotation of the apparatus (shaft) is at 0 mm.

S.11 Evaluation of trial and motion order effects on stiffness and ROM.

Table S.11.1: Linear mixed model for range of motion (unit: deg), with effect of trial, motion order and session. CI = confidence interval.

Motion	Configuration	Variable	Estimate (95% CI)	*p* value
Flexion	Passive	Intercept	32.2 (22.5 to 41.9)	< 0.001
		Trial	0.5 (-0.2 to 1.3)	0.156
		Motion	2.6 (0.8 to 4.4)	0.005
		Session	5.2 (2.4 to 8.0)	< 0.001
	Active	Intercept	33.6 (17.1 to 50.1)	0.001
		Trial	-0.9 (-1.7 to 0)	0.052
		Motion	5.8 (3.7 to 7.9)	< 0.001
		Session	-2.4 (-5.6 to 0.8)	0.135
	Seated	Intercept	65.8 (60.5 to 71.1)	< 0.001
		Trial	0.6 (0 to 1.2)	0.062
		Motion	-0.7 (-1.3 to -0.1)	0.020
		Session	-5.1 (-6.9 to -3.3)	< 0.001
Extension	Passive	Intercept	74.1 (63.5 – 84.7)	< 0.001
		Trial	0.7 (-0.3 to 1.8)	0.159
		Motion	-3.2 (-4.7 to -1.7)	< 0.001
		Session	5.1 (2.1 to 8.0)	0.001
	Active	Intercept	76.4 (64.6 – 88.2)	< 0.001
		Trial	0.7 (-0.6 to 2.0)	0.274
		Motion	-1.3 (-3.2 to 0.5)	0.142
		Session	3.7 (0.1 to 7.4)	0.043
	Seated	Intercept	75.4 (57.8 to 92.9)	< 0.001
		Trial	0.1 (-0.5 to 0.8)	0.682
		Motion	-1.0 (-2.0 to 0)	0.040
		Session	0.1 (-2.3 to 2.4)	0.963
Left lateral bending	Passive	Intercept	38.7 (30.9 to 46.5)	< 0.001
		Trial	0.5 (-0.2 to 1.3)	0.180
		Motion	-1.0 (-1.7 to -0.2)	0.016
		Session	-0.2 (-2.4 to 1.9)	0.823
	Active	Intercept	44.4 (35.7 to 53.2)	< 0.001
		Trial	0.3 (-0.4 to 1.0)	0.440
		Motion	-1.2 (-1.9 to -0.5)	0.002
		Session	-0.2 (-2.3 to 1.9)	0.830
	Seated	Intercept	42.8 (34.9 to 50.8)	< 0.001
		Trial	0.5 (-0.2 to 1.1)	0.170
		Motion	0.5 (-0.3 to 1.3)	0.213
		Session	-3.9 (-6.3 to -1.4)	0.002
Right lateral bending	Passive	Intercept	42.8 (35.5 to 52.1)	< 0.001
		Trial	0.6 (-0.1 to 1.4)	0.078
		Motion	-0.5 (-1.2 to 0.2)	0.152

		Session	-4.6 (-6.7 to -2.6)	< 0.001
	Active	Intercept	51.8 (45.1 to 58.6)	< 0.001
		Trial	0.1 (-0.3 to 0.6)	0.608
		Motion	-1.2 (-1.7 to -0.8)	< 0.001
		Session	-7.8 (-9.1 to 6.5)	< 0.001
	Seated	Intercept	41.9 (33.9 to 49.9)	< 0.001
		Trial	0.5 (-0.1 to 1.0)	0.085
		Motion	0.4 (-0.7 to 1.4)	0.477
		Session	-5.9 (-8.5 to -3.2)	< 0.001
Left axial rotation	Passive	Intercept	68.2 (48.9 to 87.6)	< 0.001
		Trial	1.8 (0.7 to 2.9)	0.002
		Motion	0.9 (-0.1 to 2.0)	0.079
		Session	-1.2 (-4.3 to 1.9)	0.438
	Active	Intercept	63.7 (49.4 to 78.0)	< 0.001
		Trial	-0.5 (-1.2 to 0.2)	0.132
		Motion	2.1 (1.4 to 2.7)	< 0.001
		Session	4.5 (2.7 to 6.4)	< 0.001
	Seated	Intercept	64.5 (53.9 to 75.1)	< 0.001
		Trial	0.8 (0.1 to 1.5)	0.019
		Motion	-0.2 (-1.5 to 1.2)	0.815
		Session	-1.6 (-4.6 to 1.4)	0.281
Right axial rotation	Passive	Intercept	74.3 (57.7 to 90.9)	< 0.001
		Trial	0.4 (-0.4 to 1.1)	0.358
		Motion	-0.3 (-0.9 to 0.3)	0.264
		Session	0 (-2.2 to 2.2)	0.971
	Active	Intercept	71.6 (59.1 to 84.2)	< 0.001
		Trial	-0.5 (-1.1 to 0.2)	0.134
		Motion	-0.3 (-0.8 to 0.1)	0.164
		Session	1.7 (-0.1 to 3.4)	0.061
	Seated	Intercept	58.0 (47.6 to 68.4)	< 0.001
		Trial	1.9 (1.3 to 2.5)	< 0.001
		Motion	1.0 (0.3 to 1.6)	0.003
		Session	-4.2 (-6.0 to -2.3)	< 0.001

Results were obtained from linear mixed models with fixed effects for trial number, motion order and session number, and a random effect for participant number.

Trial number and motion order were continuous variables; session number was a categorical variable (Session 1= 1; Session 2 = 2).

Table S11.2: Linear mixed model for stiffness (unit: Nmm/deg), with effect of trial, motion order and session. CI = confidence interval.

Motion	Zone	Variable	Estimate (95% CI)	*p* value
Flexion	1	Intercept	23 (1 to 45)	0.145
		Trial	2 (-1 to 5)	0.174
		Motion	4 (-2 to 10)	0.158
		Session	-10 (-21 to 0)	0.046
	2	Intercept	40 (12 to 69)	0.025
		Trial	-1 (-4 to 3)	0.750
		Motion	-1 (-9 to 7)	0.841
		Session	-8 (-21 to 5)	0.237
	3	Intercept	60 (5 to 115)	0.085
		Trial	7 (0 to 14)	0.041
		Motion	1 (-14 to 16)	0.933
		Session	-15 (-40 to 9)	0.221
Extension	1	Intercept	21 (6 to 37)	0.016
		Trial	-1 (-3 to 2)	0.554
		Motion	1 (-2 to 4)	0.477
		Session	-3 (-10 to 4)	0.382
	2	Intercept	12 (-9 to 32)	0.142
		Trial	2 (-1 to 5)	0.245
		Motion	2 (-2 to 6)	0.422
		Session	6 (-2 to 14)	0.128
	3	Intercept	43 (8 to 77)	0.005
		Trial	3 (-3 to 9)	0.378
		Motion	0 (-8 to 7)	0.939
		Session	15 (-2 to 32)	0.076
Left lateral bending	1	Intercept	40 (18 to 63)	< 0.001
		Trial	0 (-3 to 3)	1.000
		Motion	-1 (-4 to 3)	0.638
		Session	3 (-7 to 13)	0.546
	2	Intercept	85 (54 to 117)	< 0.001
		Trial	-1 (-5 to 3)	0.661
		Motion	-5 (-9 to -1)	0.027
		Session	7 (-5 to 18)	0.242
	3	Intercept	112 (61 to 162)	< 0.001
		Trial	3 (-3 to 9)	0.388
		Motion	-4 (-11 to 2)	0.163
		Session	32 (14 to 49)	< 0.001
Right lateral bending	1	Intercept	66 (41 to 91)	< 0.001
		Trial	-1 (-5 to 2)	0.355
		Motion	-3 (-6 to 0)	0.032
		Session	-9 (-18 to 0)	0.042

	2	Intercept	81 (36 to 126)	0.004
		Trial	3 (-1 to 7)	0.163
		Motion	-5(-9 to -1)	0.016
		Session	-12 (-24 to 1)	0.061
	3	Intercept	90 (39 to 141)	< 0.001
		Trial	1 (-6 to 8)	0.812
		Motion	5 (-2 to 11)	0.144
		Session	16 (-4 to 36)	0.121
Left axial rotation	1	Intercept	57 (21 to 93)	0.004
		Trial	1 (-3 to 4)	0.674
		Motion	-5 (-9 to -2)	0.002
		Session	4 (-6 to 14)	0.450
	2	Intercept	40 (19 to 60)	< 0.001
		Trial	2 (0 to 4)	0.038
		Motion	-1 (-3 to 1)	0.195
		Session	10 (4 to 16)	0.002
	3	Intercept	60 (36 to 85)	< 0.001
		Trial	1 (-3 to 5)	0.553
		Motion	-2 (-5 to 2)	0.406
		Session	20 (9 to 31)	< 0.001
Right axial rotation	1	Intercept	37 (7 to 67)	0.022
		Trial	1 (-1 to 2)	0.593
		Motion	0 (-2 to 1)	0.773
		Session	1 (-4 to 6)	0.765
	2	Intercept	39 (17 to 61)	< 0.001
		Trial	0 (-3 to 3)	0.939
		Motion	-1 (-3 to 1)	0.412
		Session	17 (9 to 25)	< 0.001
	3	Intercept	58 (35 to 81)	< 0.001
		Trial	1 (-2 to 4)	0.453
		Motion	-2 (-4 to 0)	0.126
		Session	25 (17 to 34)	< 0.001

Results were obtained from linear mixed models with fixed effects for trial number, motion order and session number, and a random effect for participant number.
Trial number and motion order were continuous variables; session number was a categorical variable (Session 1= 1; Session 2 = 2).

Table S.11.3: Motion order for each participant (P) in both sessions. (S1: session 1; S2: session2; F: flexion; E: extension; L: left; R: right; RL: left axial rotation; RR: right axial rotation).

**Participant**	**P1**		**P2**		**P3**		**P4**		**P5**		**P6**	
**Session**	**S1**	**S2**	**S1**	**S2**	**S1**	**S2**	**S1**	**S2**	**S1**	**S2**	**S1**	**S2**
**Seated**	F	E	RR	F	RL	RL	L	RR	F	RR	L	RL
	RR	RL	R	RR	L	RR	E	RL	L	E	F	RR
	RL	L	RL	RL	R	E	R	E	RR	RL	R	F
	L	R	L	L	E	F	RR	L	RL	L	RL	R
	R	F	E	E	F	L	F	F	E	F	RR	E
	E	RR	F	R	RR	R	RL	R	R	R	E	L
**Lying**	R	E	RL	L	E	RR	RR	R	L	RR	RR	RR
	L	F	RR	R	F	RL	RL	L	R	RL	RL	RL
	E	L	E	E	L	F	L	F	E	F	R	F
	F	R	F	F	R	E	R	E	F	E	L	E
	RR	RR	L	RL	RL	L	F	RR	RL	R	F	L
	RL	RL	R	RR	RR	R	E	RL	RR	L	E	R

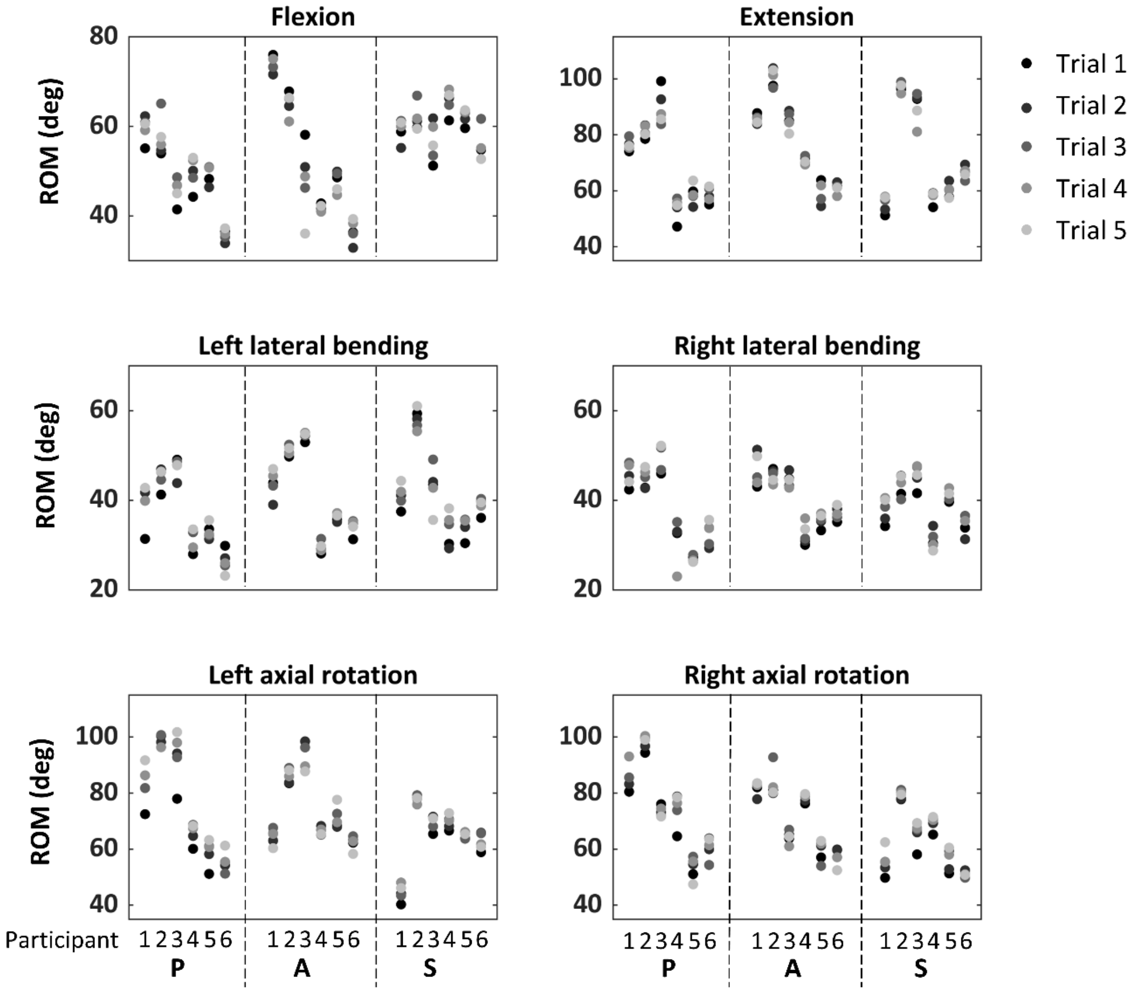

Figure S.11.1: Head-neck range of motion (ROM; P: passive-lying, A: active-lying, S: active-seated) for all participants and all trials, from session 1. Each greyscale dot represents one trial, with the black dots representing trial 1, and the lightest grey dot representing trial 5.

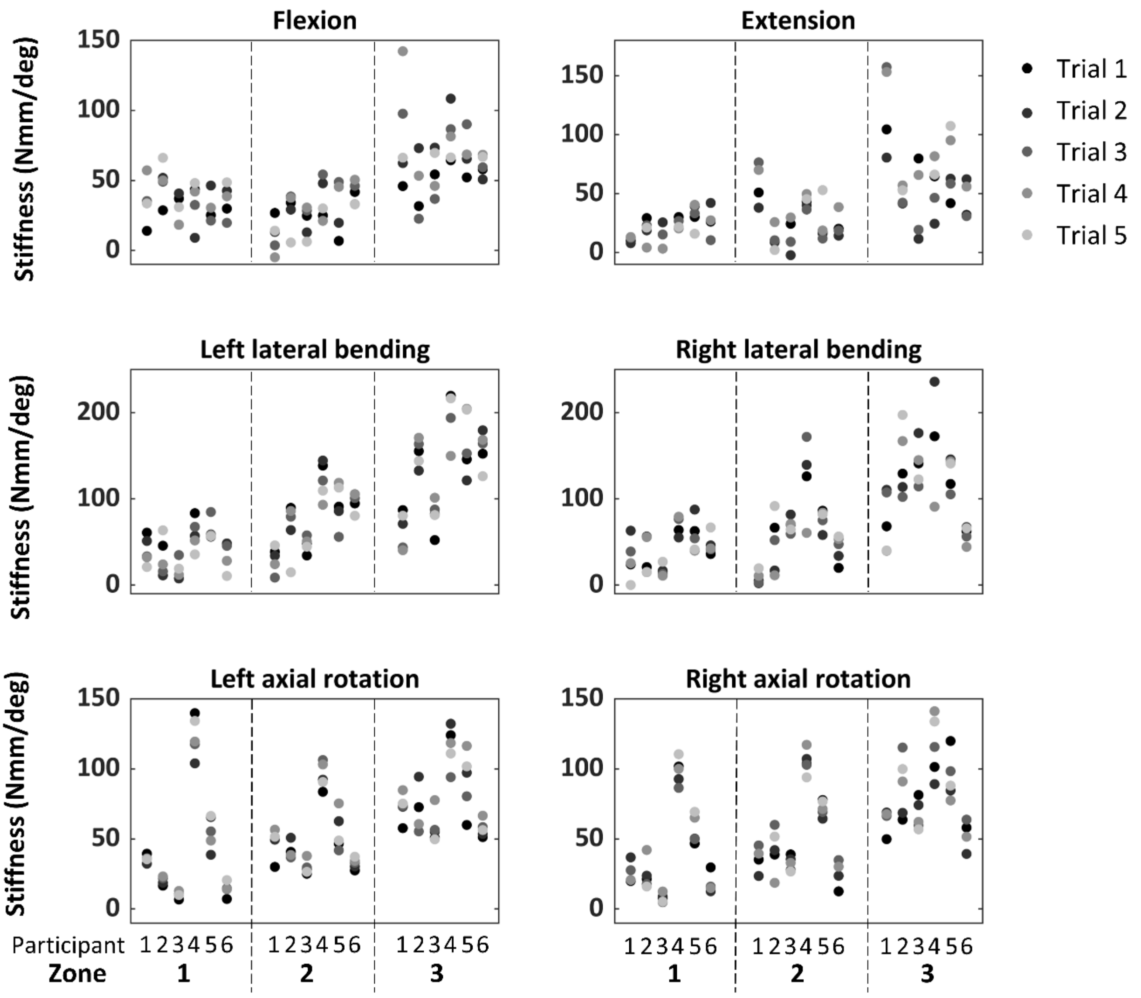

Figure S.11.2: Passive lying head-neck stiffness for all participants and all trials, from session 1. Each greyscale dot represents one trial, with the black dots representing trial 1, and the lightest grey dot representing trial 5.

S.12 Full ROM

Table S.12: Full range of motion (mean ± standard deviation; degrees) during passive tests, prior to adjustment due to muscle activation exceeding MVC threshold.

Flexion	Extension	Left lateral bending	Right lateral bending	Left axial rotation	Right axial rotation
50 ± 8	70 ± 14	37 ± 8	39 ± 9	76 ± 18	74 ± 15

S.13 Knots

Table S.13: Knot 1 (division angle between zone 1 and zone 2) and knot 2 (division angle between zone 2 and zone 3) in degrees, for each motion direction.

	Flexion	Extension	Left lateral bending	Right lateral bending	Left axial rotation	Right axial rotation
**Knot 1**	14 ± 8	24 ± 16	16 ± 7	15 ± 9	31 ±16	32 ±15
**Knot 2**	37 ± 7	53 ± 13	28 ±6	30 ±7	58 ± 14	57 ±13

S.14 Comparison of stiffness corridor from current study with results of McGill *et al.*, in flexion, extension, left and right lateral bending

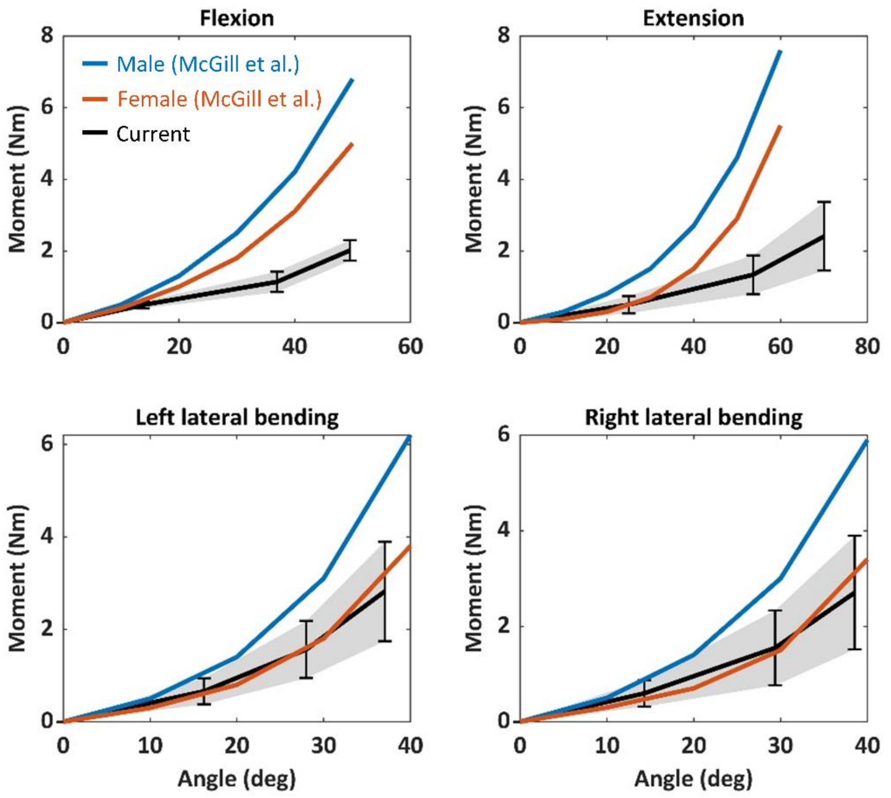

Figure S.14: Comparison of the current stiffness corridor with results obtained by McGill *et al.*[19] for flexion, extension, left and right lateral bending. McGill *et al.*'s results were plotted based on tabulated angle and bending moment data.

S.15 Comparison of stiffness from current study, with that of Dugailly et al, axial rotation

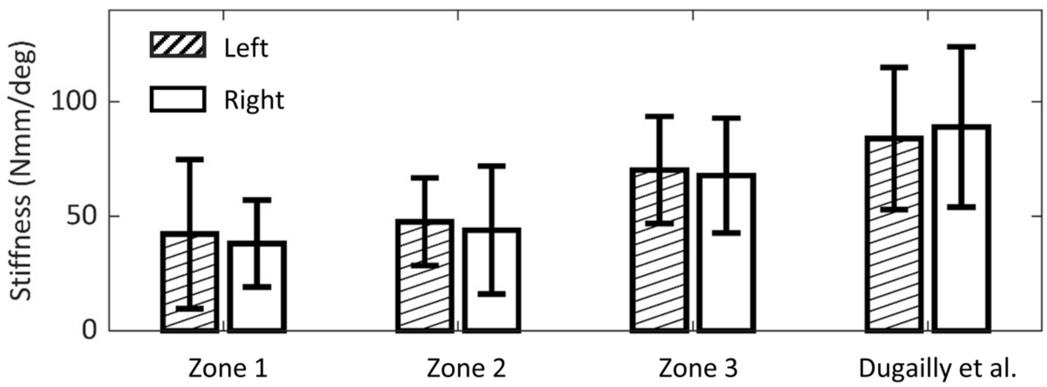

Figure S.15: Comparison of the stiffness (mean ± standard deviation) from the current study, with that obtained by Dugailly *et al.*[4] for left and right axial rotation.

S.16 Instantaneous centre of rotation for flexion, extension and lateral bending

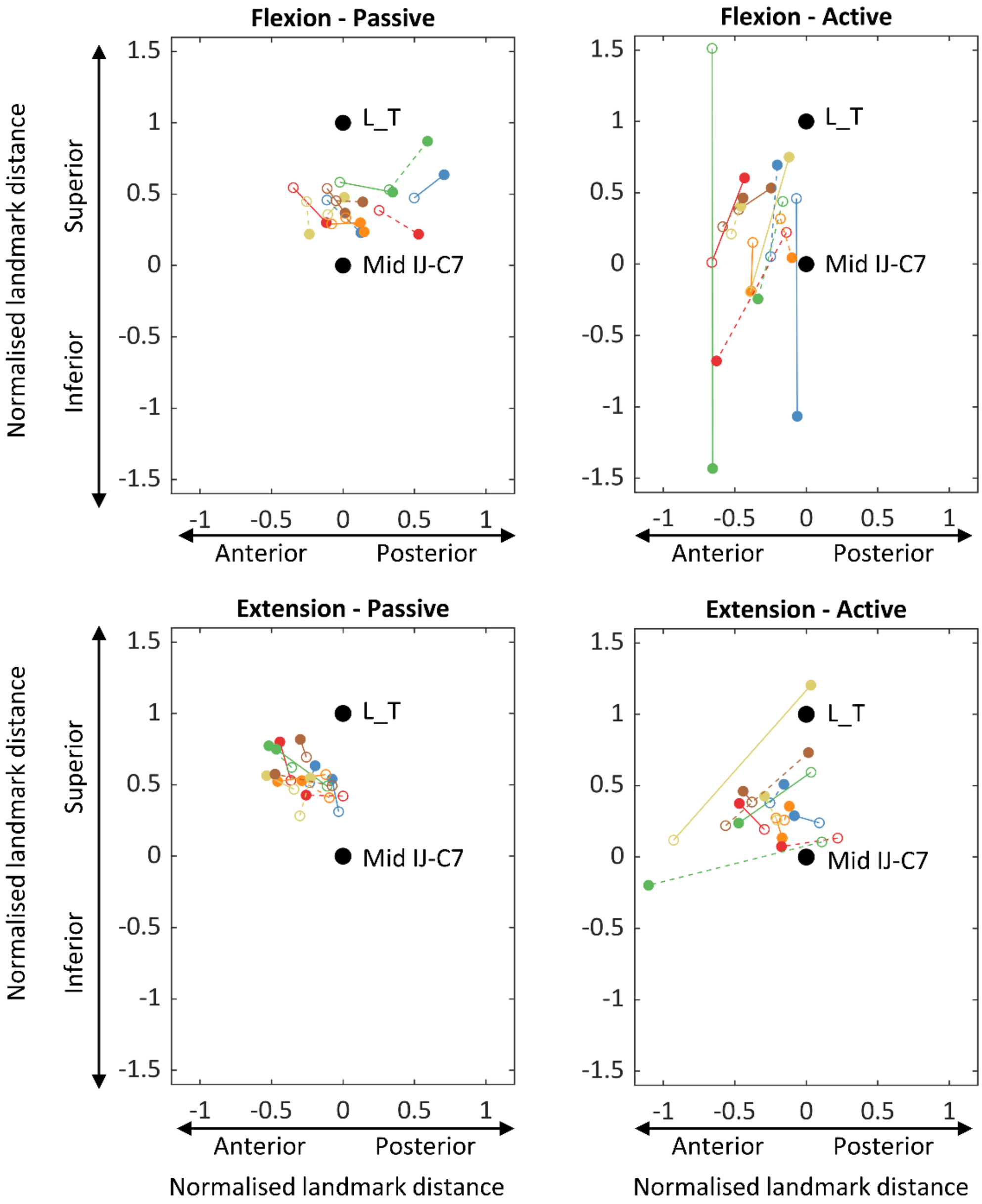

Figure S.16.1: Location of the instantaneous centre of rotation (ICR) for flexion and extension for passive and active lying tests, in the horizontal plane (parallel to the bending apparatus frame) relative to an origin located at the projection of the C7 spinous process marker onto that plane. Open circles represent the mean ICR location at neutral position, filled circles represent mean ICR location at maximum range of motion (ROM), from all trials for each participant. Lines are the linear paths of ICR from neutral to maximum ROM in session 1 (solid) and 2 (dashed). Data are normalised to an approximate “neck length”, estimated as the distance between the left tragion (L_T) and the mid-point of sternal notch (IJ) and C7 spinous process. Each colour represents one participant, consistent with all similar figures.

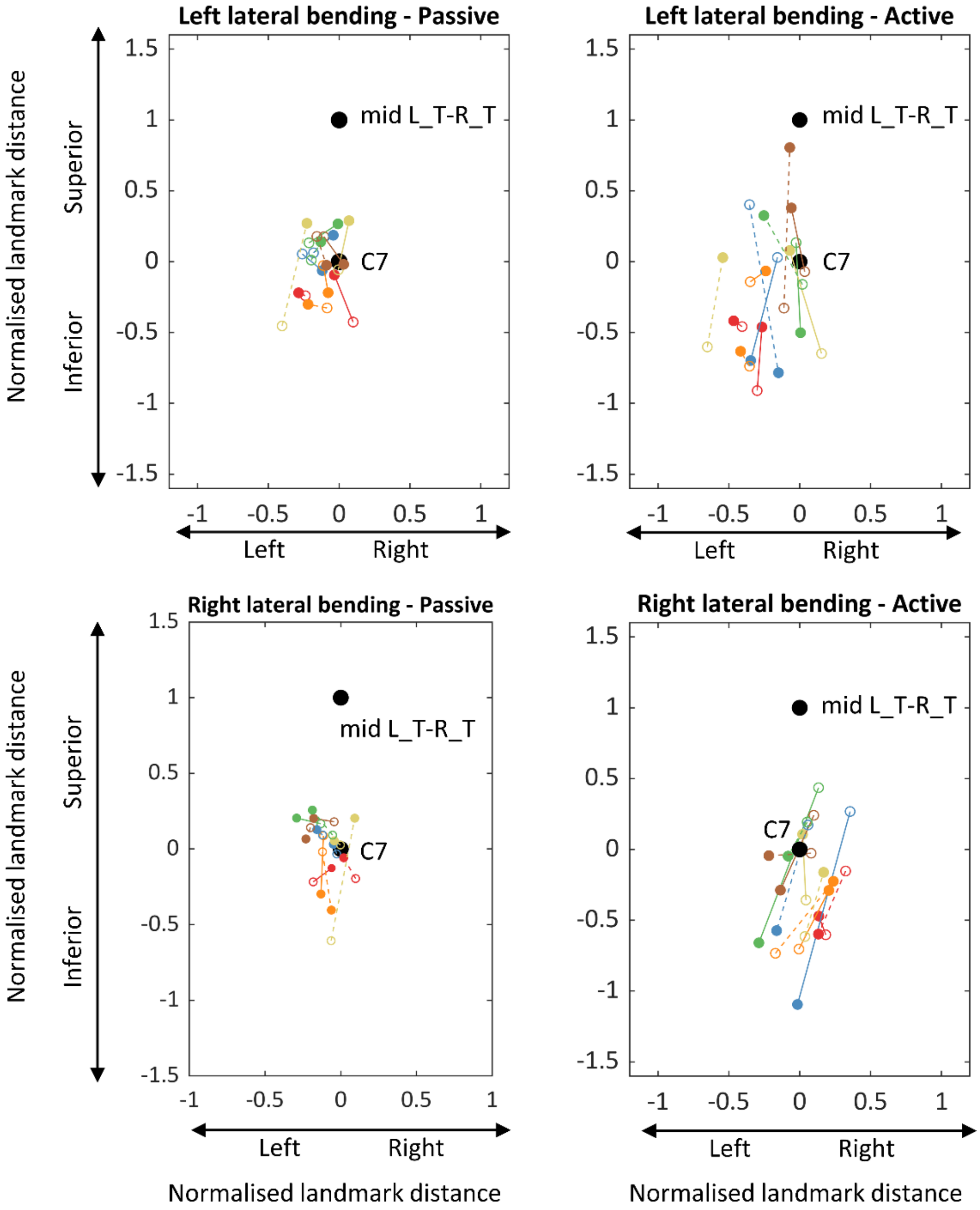

Figure S.16.2: Location of the instantaneous centre of rotation (ICR) for lateral bending, for passive and active lying tests, in the horizontal plane (parallel to the bending apparatus frame) relative to an origin located at the projection of the C7 spinous process marker onto that plane. Open circles represent the mean ICR location at neutral position, filled circles represent mean ICR location at maximum range of motion (ROM), from all trials for each participant. Lines are the linear paths of ICR from neutral to maximum ROM in session 1 (solid) and 2 (dashed). Data are normalised to an approximate “neck length”, estimated as the distance between the mid-point of the left (L_T) and right tragion (R_T) and C7 spinous process. Each colour represents one participant, consistent with all similar figures.

S.17 Rotational axis in axial rotational motion

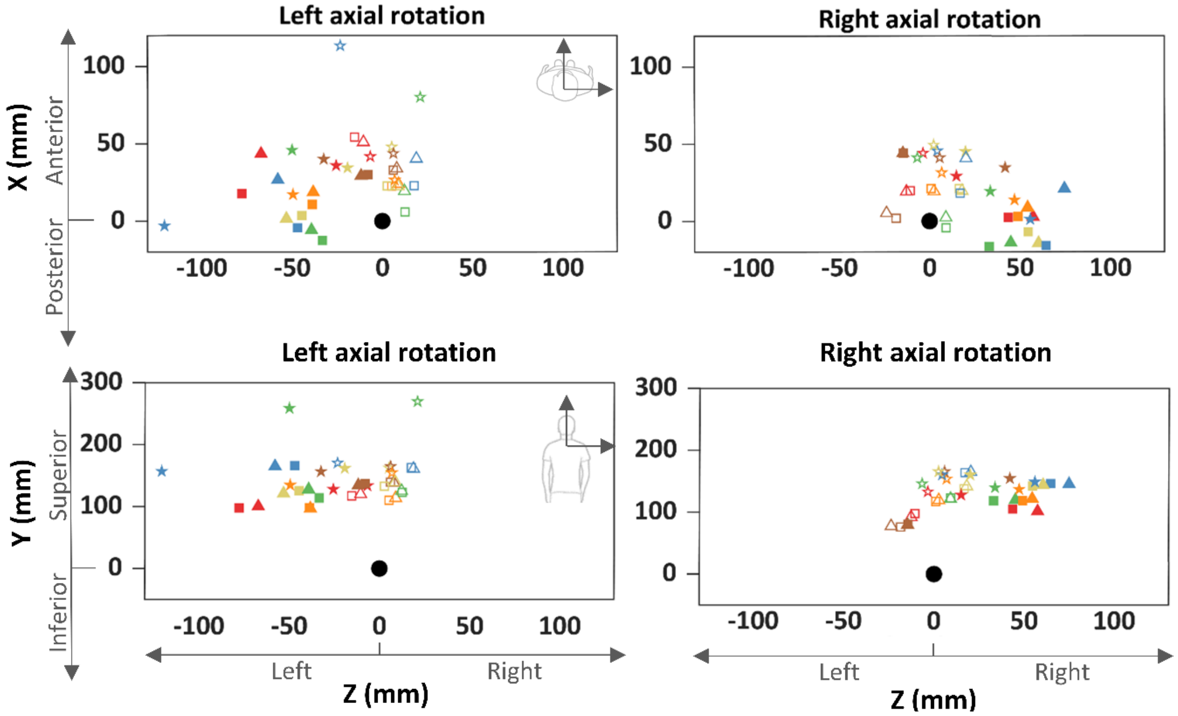

Figure S.17: Position of the mid-point of left and right tragion, relative to the mid-point of IJ-C7, in the XZ and YZ planes of the torso coordinate system (black circle at origin), from which the rotational axis was defined. X-axis: anterior-posterior axis (positive: anterior). Y-axis: superior-inferior axis (positive: superior). Z-axis: left-right axis (positive: right). Square (passive-lying), triangle (active-lying) and star (active-seated) symbols are the mean value of five trials in each configuration, at neutral (empty) and at maximum range of motion (filled). Each colour represents one participant, and is consistent with all other figures.

S.18 Method for producing stiffness "corridor" plots

Stiffness "corridor" plots were produced for each motion. Knot 1 and 2, and range of motion (ROM) were the mean values from all participants and all trials. The moment (mean ± standard deviation (S.D.)) on each knot was the corresponding angle multiplied by the stiffness (mean ± S.D.; Eq. 1). Stiffness corridor (shaded region) was defined by the stiffness value within 1 S.D. from the mean.

$$M_1 = \overline{\alpha_1} \times \overline{k_1}$$

$$M_2 = (\overline{\alpha_2} - \overline{\alpha_1}) \times \overline{k_2} + M_1$$

$$M_3 = (\overline{\alpha_3} - \overline{\alpha_2}) \times \overline{k_3} + M_2 \qquad \text{Equation (1)}$$

where: M_1, M_2, M_3 = average moment at knot 1, knot 2 and ROM

$\overline{\alpha_1}$, $\overline{\alpha_2}$, $\overline{\alpha_3}$ = average angle at knot 1, knot 2 and ROM

$\overline{k_1}$, $\overline{k_2}$, $\overline{k_3}$ = average stiffness in zone 1, zone 2 and zone 3.

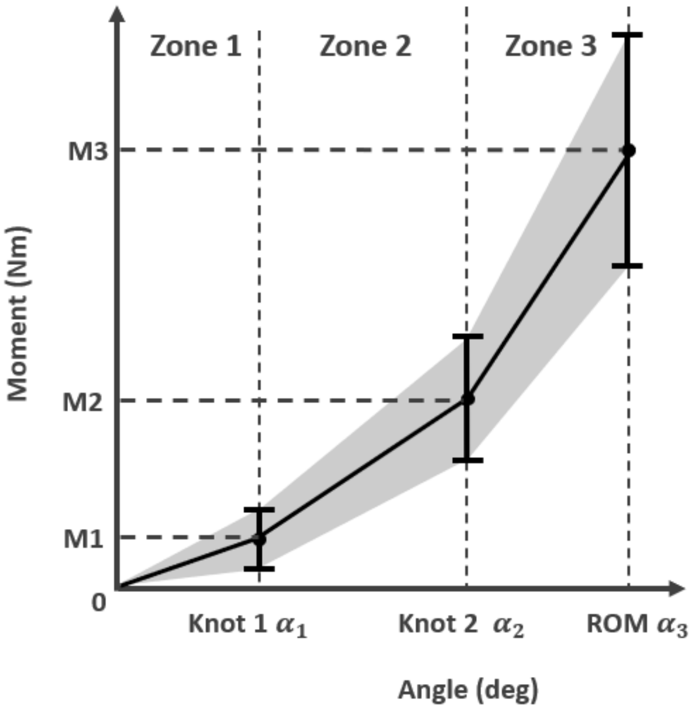

Figure S.18.1 : Exemplar stiffness corridor plot. Shaded corridor represents stiffness within 1 standard deviation $\boldsymbol{M_1}$, $\boldsymbol{M_2}$, $\boldsymbol{M_3}$, $\boldsymbol{\alpha_1}$, $\boldsymbol{\alpha_2}$ and $\boldsymbol{\alpha_3}$ define the moment and angle at each knot.

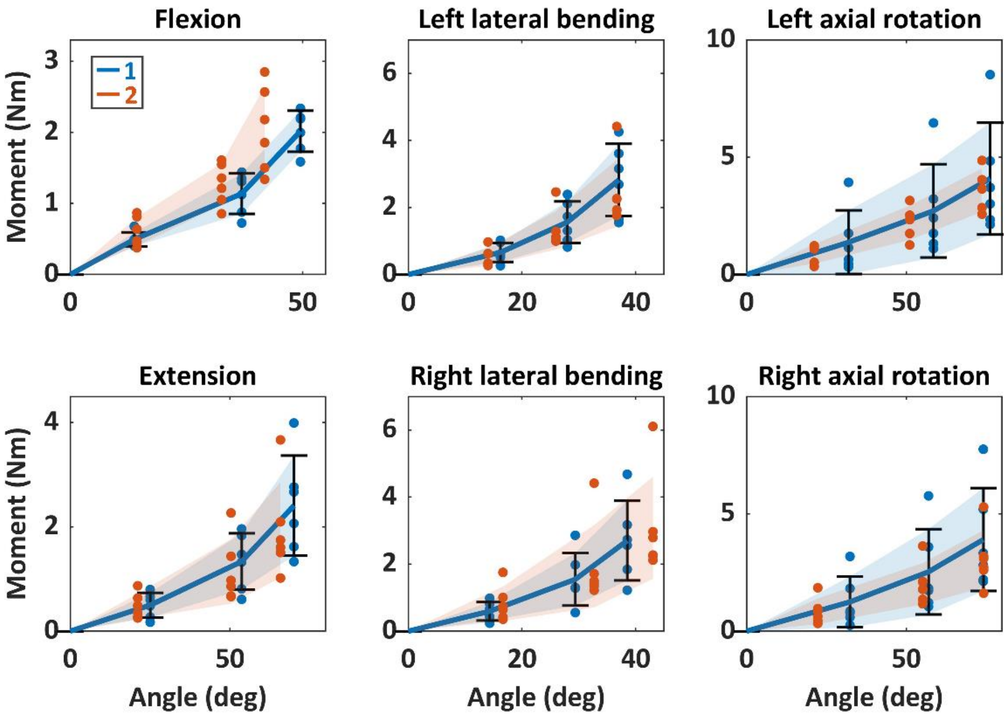

Figure S.18.2: Stiffness corridors from both sessions. Shaded regions are the stiffness corridors. Error bars represent session 1 moment that are one standard deviation away from the mean. Dots are participant's data points.
